# A transcriptional activator from *Rhizophagus irregularis* regulates phosphate uptake and homeostasis in AM symbiosis during phosphorous starvation

**DOI:** 10.3389/fmicb.2022.1114089

**Published:** 2023-01-20

**Authors:** Shuyuan Zhang, Yuying Nie, Xiaoning Fan, Wei Wei, Hui Chen, Xianan Xie, Ming Tang

**Affiliations:** State Key Laboratory of Conservation and Utilization of Subtropical Agro-Bioresources, Guangdong Laboratory for Lingnan Modern Agriculture, Guangdong Key Laboratory for Innovative Development and Utilization of Forest Plant Germplasm, College of Forestry and Landscape Architecture, South China Agricultural University, Guangzhou, China

**Keywords:** arbuscular mycorrhizal fungi, P starvation, P uptake, RiPho4, transcription factor, yeast one-hybrid, virus-induced gene silencing

## Abstract

**Introduction:**

Phosphorus (P) is one of the most important nutrient elements for plant growth and development. Under P starvation, arbuscular mycorrhizal (AM) fungi can promote phosphate (Pi) uptake and homeostasis within host plants. However, the underlying mechanisms by which AM fungal symbiont regulates the AM symbiotic Pi acquisition from soil under P starvation are largely unknown. Here, we identify a HLH domain containing transcription factor RiPho4 from *Rhizophagus irregularis*.

**Methods:**

To investigate the biological functions of the RiPho4, we combined the subcellular localization and Yeast One-Hybrid (Y1H) experiments in yeasts with gene expression and virus-induced gene silencing approach during AM symbiosis.

**Results:**

The approach during AM symbiosis. The results indicated that *RiPho4* encodes a conserved transcription factor among different fungi and is induced during the *in planta* phase. The transcription of *RiPho4* is significantly up-regulated by P starvation. The subcellular localization analysis revealed that RiPho4 is located in the nuclei of yeast cells during P starvation. Moreover, knock-down of *RiPho4* inhibits the arbuscule development and mycorrhizal Pi uptake under low Pi conditions. Importantly, RiPho4 can positively regulate the downstream components of the phosphate (PHO) pathway in *R. irregularis*.

**Discussion:**

In summary, these new findings reveal that RiPho4 acts as a transcriptional activator in AM fungus to maintain arbuscule development and regulate Pi uptake and homeostasis in the AM symbiosis during Pi starvation.

## Introduction

Arbuscular mycorrhizal fungi (AMF) belong to the Glomeromycotina in the Mucoromycota, and are a kind of the obligate soilborne fungi which can form the AM symbioses with more than 70% of land plants (Brundrett and Tedersoo, 2019; Bonfante and Venice, 2020; Genre et al., 2020; Rich et al., 2021). AMF have been shown to benefit plant productivity and they can absorb water and mineral nutrients such as phosphorus (P), nitrogen (N), iron, sulfur and zinc from soils, then transfer them to the host plants *via* the symbiotic interfaces (Watts-Williams and Cavagnaro, 2018; Kobae, 2019; Rahman et al., 2020; Ma et al., 2022). In return, host plants can transport fatty acids and sugars to AMF as the carbon and energy sources (Campos-Soriano and Segundo, 2011; Jiang et al., 2017; Luginbuehl and Oldroyd, 2017; An et al., 2019). These bidirectional processes effectively regulate the nutrient balance between the host plants and their AM fungal symbionts, and thus these associations are capable of promoting plant development and fitness (Pozo and Azcón-Aguilar, 2007; Hajong et al., 2013; Fellbaum et al., 2014).

Soil available P can be acquired at the root periphery and utilized by plants in the form of inorganic orthophosphate (Pi), however, Pi is always insufficient in the fields due to its low solubility and relative immobilization in soils (Vance et al., 2003; Hirsch et al., 2006; Nagy et al., 2009). The formation of AM symbiosis is an effective strategy for land plants to cope with low Pi availability (Cibichakravarthy et al., 2015; Dierks et al., 2021). During colonization, the branch hyphae of spores produce swellings called appressoria on the surface of the root epidermal cells after the perception of host plant-derived strigolactones (Giovannetti et al., 1993; Akiyama et al., 2005). Subsequently, the appressoria penetrate the epidermal cells to grow the intraradical hyphae assembled within the prepenetration apparatus (Genre et al., 2005, 2008, 2012; Russo et al., 2019); the developing intraradical mycelium (IRM) then run across the root cortical cells and form the tree-like structures called arbuscules in these cortical cells, where the nutrient transport and unloading (such as Pi and N) occurs (Parniske, 2008; Gutjahr and Parniske, 2013; Luginbuehl and Oldroyd, 2017; Hui et al., 2022). Meanwhile, arbuscules are surrounded by the extension of plant plasma membrane called the periarbuscular membrane (PAM) (Harrison et al., 2002; Pumplin et al., 2012). It is also considered to be the main nutrient exchange site of AM symbiosis (Balestrini and Bonfante, 2014; Ivanov and Harrison, 2014; Roth et al., 2019). During AM symbiosis, the extraradical mycelium (ERM) of AMF can reach up to 100 times length of root hairs (Javot et al., 2007) and form the large external hyphal networks to expand more area for Pi absorption beyond the rhizospheres, and also increase the phosphatase activities at the rhizospheres (Wang et al., 2016; Hu et al., 2019). Therefore, the AM symbioses can enhance plant Pi uptake and utilization during P starvation (Smith, 2009; Cibichakravarthy et al., 2015; Dierks et al., 2021).

Earlier radiotracer studies have demonstrated that Pi travels from soils through the AM fungal hyphae to the host plants (Pearson and Jakobsen, 1993; Smith et al., 2003). In past two decades, the high-affinity transporter genes belonging to the PHT1 (PHOSPHATE TRANSPORTER 1) family that are expressed in the ERM and IRM have been identified and characterized from some AMF species, for example, *GmosPT*, *GigmPT*, *GvPT*, and *RiPT* from *Glomus mosseae, Gigaspora margarita*, *Glomus versiforme* (currently *Diversispora epigaea*), and *Rhizophagus irregularis* (Harrison and van Buuren, 1995; Maldonado-Mendoza et al., 2001; Benedetto et al., 2005; Fiorilli et al., 2013; Xie et al., 2016; Sun et al., 2019; Venice et al., 2020). AMF can absorb Pi from soil by ERM, and polymerize Pi into polyphosphate (Poly-P) by vacuolar transporter chaperone (VTC) complex; the Poly-P was accumulated in the vacuoles, and then transferred to the IRM associating with water transport process (Kikuchi et al., 2016). The Poly-P phosphatases Ppn1 and Ppx1 in IRM can hydrolyze the Poly-P into Pi, and export it from vacuoles to the cytoplasm through the unknown P transporters located in the vacuole membrane (Solaiman et al., 1999; Ezawa and Saito, 2018; Xie et al., 2022).

It has been shown that there is a specialized Pi export system in the arbuscules, where free Pi is transported and unloaded into the periarbuscular space (PAS) (Ezawa and Saito, 2018; Zhou et al., 2021; Xie et al., 2022). After the Poly-P hydrolyzation in the IRM and arbuscules, the Pi transporters containing SPX (SYG1/Pho81/XPR1) domains participate in the Pi export process at the symbiotic interface, releasing Pi into the PAS (Ezawa and Saito, 2018; Plassard et al., 2019; Xie et al., 2022). Pi in the PAS cross the PAM to root cortical cells relies on the mycorrhiza-induced phosphate transporters belonging to the plant PHT1 gene family (Javot et al., 2007; Yang et al., 2012; Xie et al., 2013; Volpe et al., 2016). On the other hand, it has been found that AMF also possesses the low-affinity Pi transport system (such as Pho87/90/91) and phosphatases (Tisserant et al., 2012; Lin et al., 2014; Venice et al., 2020). The Pi transport systems in AMF is very similar to that of *Saccharomyces cerevisiae*, which is well-known to contain the high-affinity system Pho84p and Pho89p and the low-affinity system Pho87p, Pho90p, and Pho91p (Auesukaree et al., 2003). This suggests that there exists a conserved PHOSPHATE (PHO) signaling pathway between AMF and yeasts (Aono et al., 2004; Olsson et al., 2006; Kikuchi et al., 2014; Zhou et al., 2021).

Transcription factors (TFs) play crucial roles in the regulation of gene expression in fungal cells and determine the functions of eukaryotic cells (Shelest, 2008). Recent advances have been made in identifying several hub TFs in mycorrhizal plants functioning in the control of AM symbiosis nutrient uptake and exchange (Pimprikar and Gutjahr, 2018; Shi et al., 2021; Das et al., 2022; Ho-Plágaro and García-Garrido, 2022). By contrast, the studies on AM fungal TFs are very limited. In two previous studies, only a few TFs, such as RiMsn2 from *R. irregularis* and GintSTE from *Glomus intraradices* (currently *R. irregularis*) are preliminarily investigated (Tollot et al., 2009; Sun et al., 2018), while the key transcriptional regulators engaged in Pi absorption and homeostasis have not been explored in AMF. Some recent studies have shown that there exists the bHLH domain-containing transcription factors encoding genes involved in the PHO pathway in response to low Pi conditions in *G. margarita*, *Gigaspora rosea* and *R. irregularis* (Tang et al., 2016; Xie et al., 2016, 2022; Zhou et al., 2021). It is well-known that in *S. cerevisiae*, the AMF bHLH ortholog ScPho4 is the transcription factor regulating the PHO pathway to control Pi absorption and homeostasis (Lenburg and O’Shea, 1996; Auesukaree et al., 2004; Wykoff et al., 2007). Although several studies have revealed that some important genes are involved in the Pi signaling and metabolism pathways in AM fungal symbionts (Balestrini et al., 2007; Xie et al., 2016, 2022), the underlying molecular mechanisms on the regulation of Pi uptake and homeostasis in AMF during symbiosis remain elusive.

*Eucalyptus* species is the most valuable and widely planted hardwood in the world (Qin et al., 2021). It has many advantages such as fast growth and strong adaptability to drought, fire, insect pest, soil acidity and low fertility (Rockwood et al., 2008). *Eucalyptus* wood can be used as an important raw material for industrial pulp and paper making, fuel and charcoal production because of its high-density property (Rockwood et al., 2008; Kato and Hibino, 2009). Because of its economic and ecological values, it is important to enhance the productivity of *Eucalyptus* limited by environmental factors such as P and N (Santos et al., 2016; Yao et al., 2021; Che et al., 2022). AM symbiosis is an environmentally friendly strategy to promote the *Eucalyptus* plants nutrient absorption when compared with fertilizer excessive use (Smethurst, 2010). Recently, there are many researches on physiological roles of AMF and ectomycorrhizal fungi on the *Eucalyptus* plants (Pagano and Scotti, 2008; Santos et al., 2021), but little studies focus on the regulatory mechanisms of Pi uptake and exchange processes in AM fungal symbiont during AM fungus-*Eucalyptus* symbiosis.

After the investigation of Pi uptake and transport processes during AMF and plant interaction, to further understand the regulatory mechanisms of the Pi uptake and homeostasis at the symbiotic interface, we start to search the regulators (TFs) in *R. irregularis* expressed during the *in planta* phase. Here, we show a new transcription factor from *R. irregularis* (RiPho4), which contains a C-terminal bHLH domain, and provide experimental evidence for roles in the regulation of Pi uptake and homeostasis during AMF-*Eucalyptus* symbiosis. Moreover, our findings offer new insights into the control of Pi uptake and metabolism in the AM fungal symbionts at the symbiotic interfaces.

## Materials and methods

### AM fungus and plant materials and growth conditions

AM fungus used in this study was *R. irregularis* DAOM 197198, which was propagated in the pot cultures with maize (*Zea mays*). Spores of *R. irregularis* were collected from *Z. mays* root segments. The plant material used in this study was *Eucalyptus grandis* (The seeds was from the Research Institute of Tropical Forestry, Chinese Academy of Forestry). The surface-sterilization of *E. grandis* seeds was performed as the described previously (Plasencia et al., 2016). The seedlings germinated were transferred from solid medium to the pots, and then inoculated with *R. irregularis* (about 200 spores per plant). After inoculation, *E. grandis* plants are cultivated in a growth chamber under 16 h: 8 h, 24°C: 19°C, light: dark conditions (light intensity, 100–200 Wm^−2^; relative humidity, 55%).

### Phosphate treatment

*Eucalyptus grandis* plants were cultivated in pots under AMF inoculation (AM) and uninoculated control (NM) treatments. Each treatment was carried out with three Pi concentrations including 30, 300 and 1,000 μM K_2_HPO_4_ (Sugimura and Saito, 2017; Fan et al., 2020). The plants were fertilized once a week using the modified Long-Ashton (mLA) solutions (Hewitt, 1966) containing the indicated Pi concentrations. After 45 days treatments, NM and AM plants were collected and stored at-80°C for subsequent experiments. And plant roots colonized with AMF in the pot experiment were collected to extract DNA and RNA from *R. irregularis*.

For RiPho4 subcellular localization analysis, yeast cells were cultured in YNB medium without uracil (Ura) for 24 h. And the yeast cells were cultured in YNB/-Ura medium for 12 h, supplemented with different Pi concentrations (the final Pi concentrations in each were 600 μM, 1 mM, 10 mM KH_2_PO_4_, respectively) (O’Neill et al., 1996; Komeili and O’Shea, 1999; Zhou and O’Shea, 2011).

### Genomic and RNA-seq data analysis

To identify the candidate genes of the PHO pathway in *R. irregularis*, the genes of PHO signaling pathway in *S. cerevisiae* was used as the queries to search for the homologues genes in the genome of *R. irregularis* DAOM 197198 (Supplementary Table S2). Through genome BLAST in NCBI and GEO databases, tBLASTn and BLASTp searches were carried out to search the target genes in *R. irregularis*, and the best matching sequence ranking first from BLASTp results is used for subsequent analysis. The homologous amino acid sequences were collected from the NCBI database.

To analyze the expression profiles of the target genes in different fungal tissues of *R. irregularis*, the original RNA-seq sequences of non-symbiotic tissues (germinating spores) and symbiotic tissues (mycorrhizal roots) of *R. irregularis* download from DDBJ database. The accession numbers of RNA-seq reads are as follows: germinating spores harvested at a week after inoculation (DRA002591), germinating spores collected at 7 days after induction (GSE67913), laser microdissected cells contain IRM and arbuscules collected from *Medicago truncatula* mycorrhizal roots (GSE99655), mycorrhizal roots of *M. truncatula* (GSE99655, GSE67926), ERM collected from carrot root culture (GSE99655) (Zeng et al., 2018, 2020).

### Gene expression analysis

The extraction of the genomic DNA from *R. irregularis* was referred to the method of Zézé et al. (1994) for amplification of gene fragments containing non-coding regions. Besides, total RNA was extracted by Trizol (Invitrogen) method, and the concentration and purity of total RNA were detected by NanoDrop 2000 (Thermo Scientific, United States). First-strand cDNA was produced from total RNA by a Hiscript III reverse transcriptase kit with gDNA wiper (Vazyme Biotech, Nanjing, China) following the manufacturer’s instructions. The qRT-PCR experiments were performed in a 96-well Real time PCR system instrument (BioRed, Hercules, CA, United States) (Xie et al., 2022). *RiEF1α* gene from *R. irregularis* was used as an internal control for qRT-PCR analysis. Relative expression levels were calculated using 2^−ΔΔCt^ method. The list of gene-specific primers used for qRT-PCR analysis is given in Supplementary Table S3.

### Yeast manipulations

The full-length of *RiPho4* was amplified by gene-specific primers containing the *BamH*I site (Primer sequences are listed in Supplementary Table S4). One step cloning Kit (Vazyme Biotech, Nanjing, China) was used to recombine the *RiPho4* cDNA into the pUG36 vector, and the resulting plasmid pUG36-GFP-RiPho4 was transformed into the yeast EY57 strain using the LiOAc/PEG-based method described previously (Gietz and Schiestl, 2007). Positive transformants were grown in YNB liquid medium lacking Ura for oscillation culture at 28°C for 24 h.

The ORF of *RiPho4* was cloned from cDNA of *R. irregularis* using the primers ADRiPho4-F/R, and then cloned into the pGADT7 (Chien et al., 1991). To construct bait-specific pAbAi vector, *cis*-acting elements with their flanking nucleotides from the promoters of target genes (Tomar and Sinha, 2014) (listed in Supplementary Table S4) were synthesized and cloned into the *Sal*I site of the pAbAi vector (Clontech Laboratories, United States). Then the constructed vectors were transformed into the Y1HGold yeast strain, and grown on YNB/-Ura solid medium with Aureobasidin A (AbA) concentration (100 mM) for testing the minimal inhibitory concentration of AbA for bait-specific pAbAi plasmids. We transformed the bait-specific pAbAi fragments and pGADT7-RiPho4 were co-transformed into yeast cells, and screened using the SD medium lacking Leucine. The yeast cells (OD_600_ = 0.2) containing both RiPho4-pGADT7 and promoter fragments were inoculated on SD medium lacking Leucine with different AbA concentrations (100–800 ng/ml). The yeast cells carrying pAbAi-p53 and pGADT7-SV40 were used as the positive control, whereas the yeast cells containing pGADT7-RiPho4 and promoter fragments lacking the *cis*-element served as the negative control. In addition, the inhibitory effect was adjusted based on the growth of yeast (Zhan et al., 2017; Sun et al., 2018; Yang et al., 2019).

### Virus-induced gene silencing

Tobacco (*Nicotiana benthamiana*) is used for this virus-induced gene silencing (VIGS) experiment (Zhang and Liu, 2014; Kikuchi et al., 2016; Xie et al., 2022). Two specific cDNA fragments of *RiPho4* from the-9 to +226 regions (*RiPho4-RNAi-1*) and + 1,200 to +1,429 regions (*RiPho4-RNAi-2*) relative to the start codon ATG (Supplementary Figure S5) were amplified by PCR following the method described by Senthil-Kumar and Mysore (2014). The cloned gene fragments (the primers VigsRiPho4-F1/R1 and VigsRiPho4-F2/R2 were listed in Supplementary Table S4) were ligated to the pTRV2 vector. And the resulting plasmids *pTRV2-RiPho4-1* and *pTRV2-RiPho4-2* were separately transformed into *Agrobacterium tumefaciens* GV3101 (Grønlund et al., 2013; Zhang and Liu, 2014). *A. tumefaciens* culture (OD_600_ = 1.0) with pTRV1 and that with pTRV2 or pTRV2-RiPho4 were mixed together and activated by adding 10 mM Acetosyringone, then stood in darkness for 2 h. The inoculums were injected into the leaves of *N. benthamiana*, whose roots had been inoculated with *R. irregularis* for 4 weeks as described by Kikuchi et al. (2016). Tobacco plants treated with three Pi concentrations (30 μM, 100 μM, 300 μM) were cultured in a small chamber for 2 weeks.

### AM phenotypical analysis

The collected *E. grandis* roots were digested in 10% KOH solution at 90°C for 40 min for 3 times, and washed with distilled water, then neutralized in 2% HCI solution for 20 min. After washing with sterile water for 3 times, AM roots were stained with 5 μg/ml wheat germ agglutinin 488 (WGA488, Invitrogen) at 37°C for 30 min (Phillips and Hayman, 1970; Ivashuta et al., 2005). The enzyme activities of succinate dehydrogenase (SDH), alkaline phosphatase (ALP) and acid phosphatase (ACP) were performed as described previously (Zhao et al., 1997). Mycorrhizal colonization was estimated according to Trouvelot et al. (1986).

### Microscopy

The fluorescent signals in yeast cells and AM roots were observed by a fluorescence microscope (Y-TV55; Nikon, Tokyo, Japan). The colonization levels of SDH, ACP and ALP enzyme activity staining was calculated under the light microscope (Y-TV55; Nikon, Tokyo, Japan).

### Total P concentration analysis

Fresh samples of *E. grandis* were lyophilized for 6 h. To grind dried samples into powder, we added magnetic beads to them and grinded them with a grinder (35 Hz) for 2 min. 0.3 g of sample powders were digested by 6 M nitric acid under the water bath at 90°C for 1 h (Fan et al., 2020). The digested samples were filtered with filter membranes and diluted with 5% nitric acid to 10 ml. Total P concentrations in the *E. grandis* digests were measured by the inductively coupled plasma optical emission spectrometry (ICP-OES; Varian, United States). Total P concentrations of *N. benthamiana* were detected by the tissue total P content detection Kit (Cat. NO. BC2855, Solarbio, China) and measured with the Microplate Reader (Vaioskan, Thermo Scientific, United States).

### Bioinformatics

The BLASTP^1^ was used to search homologs of RiPho4 protein in the fungi species. The characteristics of the secondary structure of RiPho4 were analyzed by the SMART program.^2^ The SWISS-MODEL website^3^ was employed to build the three-dimensional model of RiPho4. The conserved regions of RiPho4 and its homologous proteins were analyzed by Meme.^4^ The heat diagram for PHO pathway gene expression levels in different fungal tissues was made by the TBtools (Chen et al., 2020). The existence of *cis*-acting element by searching for 1.5 kb promoter sequences of the target gene coding regions through the NCBI database and PlantCARE software.^5^

### Phylogenetic analysis

The unrooted phylogenetic tree of RiPho4 protein and other amino acid sequences in different fungi species were constructed with MEGA7.0 (Kumar et al., 2016) using the neighbor-joining method. Accession numbers of all the fungal proteins are shown in Supplementary Table S2.

### Statistical analysis

Data are preliminarily accounted by Microsoft EXCEL 2016, and statistical significances between treatments were analyzed by analysis of variance (ANOVA) using SPSS software (Version 26.0, SPSS Inc., United States). The Duncan’s multiple range test were used for comparing more than two datasets. A value of *p* < 0.05 was considered to be statistically significant. The different letters indicate significant differences among phenotypes or treatments. Huang and Freiser (1993, Origin Lab, United States) to plot and illustrate diagram for changing curves of different treatments.

### Accession numbers

Sequence data from this article can be found in the AM fungal genome and GenBank libraries under the following accession numbers: RiPho4 (XP_025175129.1), RdPho4 (RGB28534.1), RcPho4 (GBB98521.1), GcPho4 (RIA86088.1), GmPho4 (KAF0357978.1), GrPho4 (RIB17793.1), DePho4 (RHZ83467.1). Other accession numbers of fungal Pho4 proteins were provided in Supplementary Table S2.

## Results

### Effects of AMF on the growth and Pi uptake of *Eucalyptus grandis* in roots under Pi-deficient conditions

**Figure 1:**
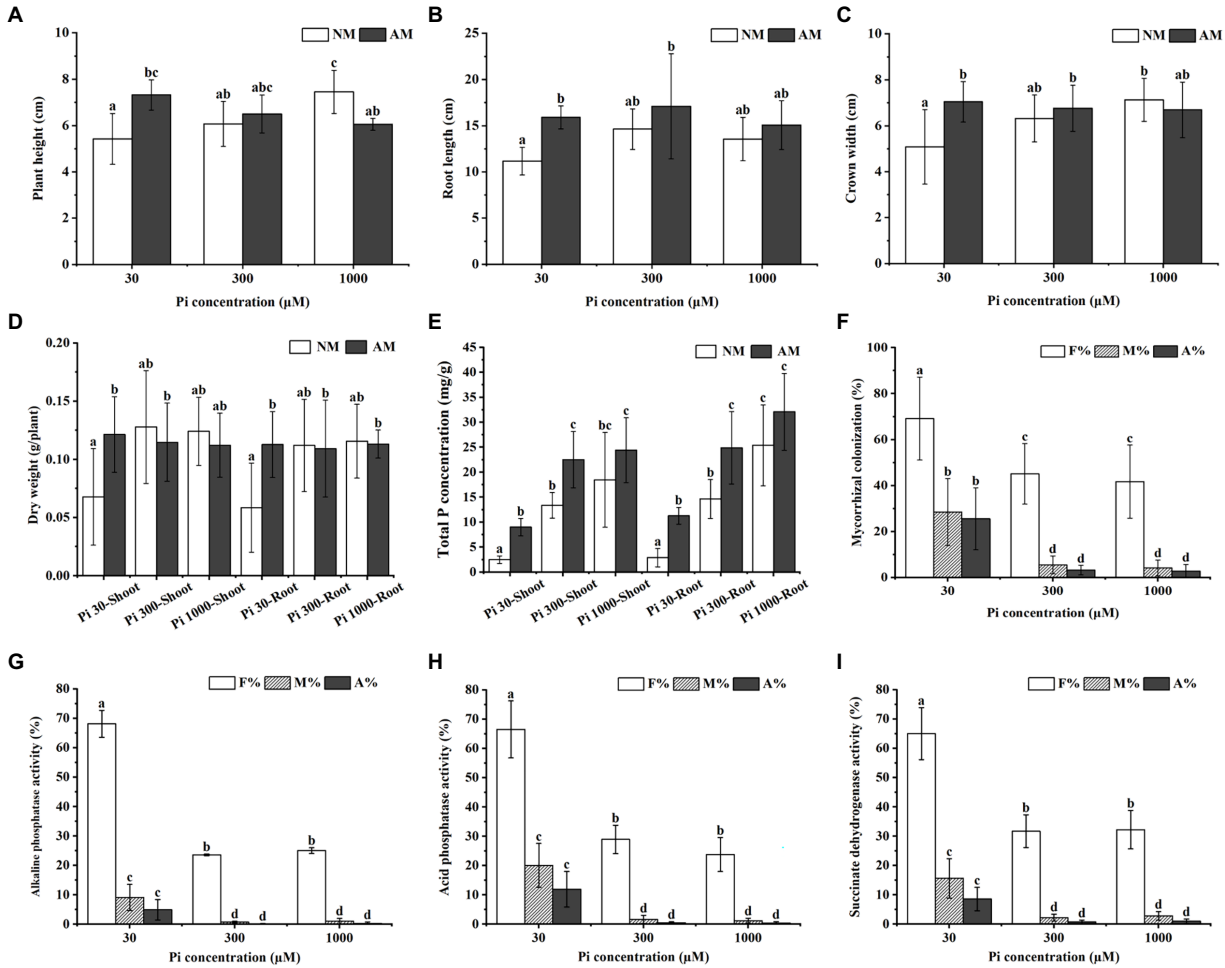
Impact of different Pi concentrations and AMF colonization on *Eucalyptus grandis* growth and enzyme activities within mycorrhizal roots. **(A–D)** The growth performances of NM and AM *E. grandis* plants under different phosphate concentrations. **(E)** Total P concentrations of shoots and roots of NM and AM *E. grandis* with *R. irregularis* grown at different Pi levels. **(F)** Mycorrhizal colonization levels among different phosphate concentrations were determined in roots after the WGA488 staining. **(G–J)** Impact of phosphate concentrations on three enzymes activities, including alkaline phosphatase (ALP), acid phosphatase (ACP) and succinate dehydrogenase (SDH), which are involved in AM fungal metabolism and Pi homeostasis during symbiosis. Data on the pictures are obtained by the ALP **(G)**, ACP **(H)**, and SDH **(I)** activities staining, respectively. (F–I) F%, the total colonization frequency; M%, the percentage of mycorrhizal intensity; A%, the percentage of arbuscule abundance. Error bars represent the mean of five biological replicates ± SD. Averages with the different letters on the top of the column mean significantly differences at *p* < 0.05 level, based on Duncan’s new multiple range test.

To study the effects of AMF inoculation on the growth and Pi uptake of *E. grandis* subjected to different Pi conditions, we treated mycorrhizal (AM) *E. grandis* with three phosphate concentrations when compared with non-mycorrhizal (NM) *E. grandis*. After 7 weeks inoculation, the growth performance of AM *E. grandis* was better than NM plants under medium and low phosphate conditions (30 and 300 μM) (Supplementary Figure S1). Overall growth (such as plant height, root length, and biomass) also showed such a trend (Figures 1A–D). Compared with NM plants, the total P concentrations of both roots and shoots showed significant increases in AM plants under low and medium phosphate concentrations (30 and 300 μM), while there were no significant differences between AM and NM plants under high Pi conditions (Figure 1E). In addition, it was found that the colonization levels of *E. grandis* exposed to low Pi (30 μM) were significantly higher than those grown under the medium and high Pi treatments (300 and 1,000 μM) (Figure 1F). Moreover, we also detected the activities of ACP, ALP and SDH involved in AM fungal function and mycorrhizal Pi utilization efficiency (Guillemin et al., 1995; Vivas et al., 2003; Kouas et al., 2009; Li et al., 2017). Activities of these three enzymes in roots exposed to 30 μM Pi were significantly higher than that subjected to 300 μM and 1,000 μM Pi (Figures 1G–I; Supplementary Table S1). Taken together, these results revealed that, during AM symbiosis, AM fungus *R. irregularis* can promote the *E. grandis* Pi uptake and utilization efficiency to improve the plant growth under Pi-deficient conditions.

### Transcription levels of PHO pathway genes in *Rhizophagus irregularis* are dependent on Pi availability

**Figure 2:**
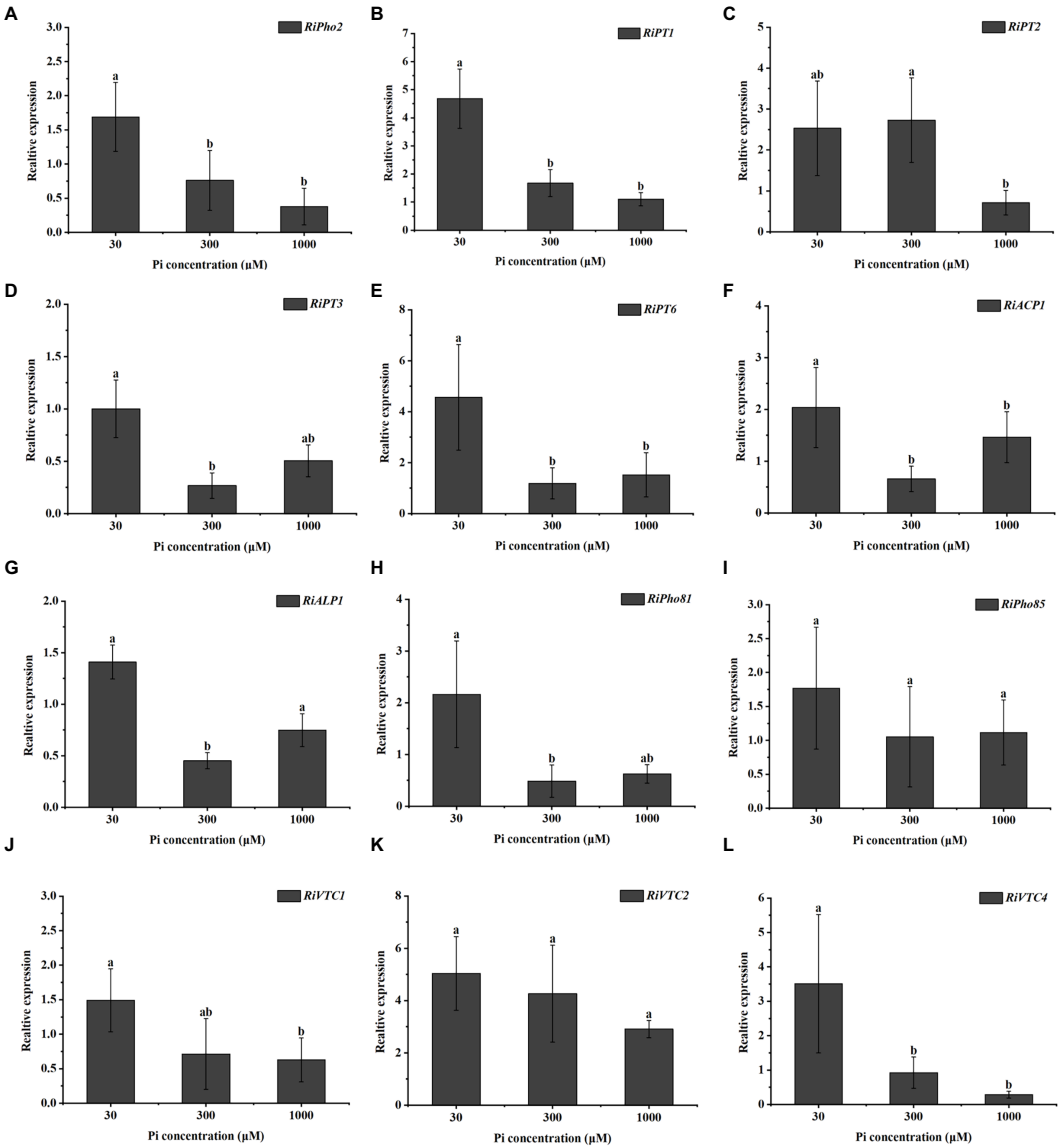
Expression profiles of the genes involved in the phosphate (PHO) signaling pathway from *R. irregularis* in mycorrhizal *E. grandis* roots at different phosphate concentrations. **(A–L)** Expression levels of genes of PHO pathway under different phosphate concentrations, including **(A)** transcription cofactor, **(B–E)** Pi transporters, **(F,G)** phosphatase related genes, **(H,I)** cyclin-protein genes and **(J–L)** vacuolar transporters, suggesting that the expressions of PHO pathway genes are affected by phosphate concentrations. AM fungal *RiEF1α* is set as the reference gene. The data represent the means of three biological replicates with standard deviations. Different letters indicate the Duncan’s multiple comparison results. Significance, *p* < 0.05.

To investigate the effect of external phosphate concentrations on the transcription levels of AM fungal genes involved in Pi uptake and metabolism, we examined the expression profiles of 12 genes in the PHO pathway of *R. irregularis* in mycorrhizal *E. grandis* roots during different Pi conditions. As shown in Figure 2A, relative to medium and high P concentrations (300 and 1,000 μM) conditions, the transcript of *RiPho2*, which was predicted to be a cofactor for RiPho4 (Chen et al., 2018; Xie et al., 2022), was significantly higher in mycorrhizal roots under low Pi (30 μM) conditions. Similarly, the expression levels of *RiPT1*, *RiPT2*, *RiPT3* and *RiPT6*, which encode the potential phosphate transporters responsible for Pi uptake, were much higher in mycorrhizal roots during low Pi concentration when compared with high Pi treatments (Figures 2B–E). Moreover, the *RiACP1* and *RiALP1* involved in Pi and Poly-P metabolisms were expressed in response to the low Pi application (Figures 2F,G). Besides, the expressions of key genes in response to Pi starvation signaling, such as *RiPho81* and *RiPho85* were also detected in mycorrhizal roots under such conditions. *RiPho81* was significantly induced at low Pi concentration, while transcripts of *RiPho85* were slightly but not significantly reduced at high Pi treatments (Figures 2H,I). On the other hand, the expressions of Poly-P accumulation-related genes *RiVTC1* and *RiVTC4* were significantly higher at 30 μM Pi than medium and high Pi concentrations (300 and 1,000 μM), whereas *RiVTC2* expression was not significantly enhanced in roots exposed to low Pi concentration (Figures 2J–L). Overall, these gene expression profiles revealed that the Pi sensing and transport/metabolism genes are regulated in response to Pi starvation in *R. irregularis* during AM symbiosis.

### Identification of RiPho4, which encodes a HLH domain-containing transcription factor

**Figure 3:**
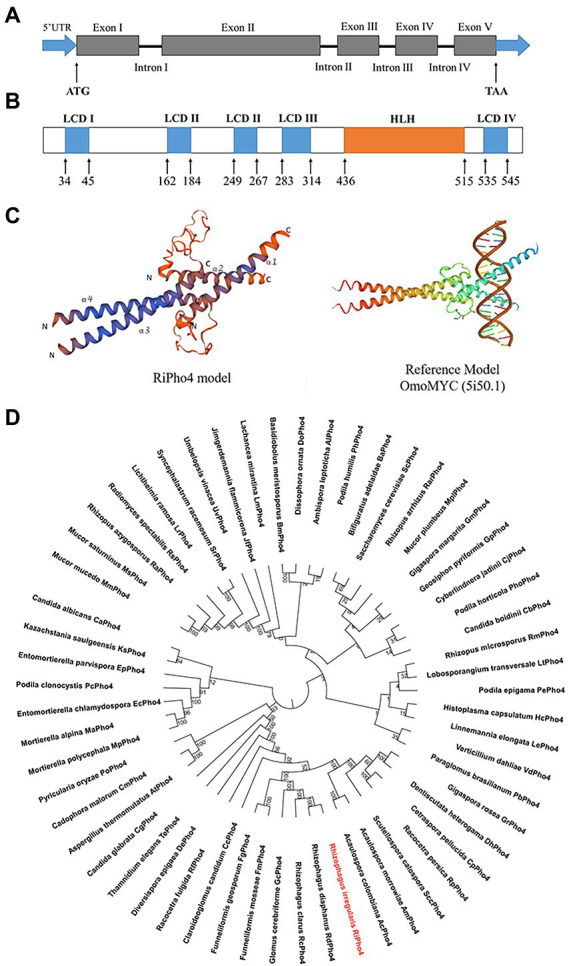
Gene and protein structures of *RiPho4* in *R. irregularis* and the evolutionary relationships with Pho4 proteins in various fungi species. **(A)** The *RiPho4* gene contains five exons and four introns, corresponding to gene ID 36869193. The untranslated regions (UTRs) are shown in the figure, ATG and TAA represent the translation start site and translation termination site, respectively. **(B)** Inferred protein domain of RiPho4. RiPho4 protein contains 570 amino acids and consists of one HLH domain and four low complex domains (LCDs) (http://smart.embl.de/). **(C)** Predicted three-dimensional structure of RiPho4 model by SWISS-MODEL. It was predicted that the 3D structure of RiPho4 is similar to that of OmoMYC (5i50.1), a protein encoded by MYC dominant-negative allele in *Mus musculus* (Jung et al., 2017). **(D)** The unrooted phylogenetic tree was constructed with the neighbor-joining method, based on multiple sequence alignments of RiPho4 (red color) and other Pho4 proteins from different fungi species using MEGA v7.0 software. Bootstrap tests were performed using 1,000 replicates. The accession numbers of all Pho4 proteins are provided in the Supplementary Table S2.

The above results (see Figure 2) indicate that the downstream genes of the PHO pathway in *R. irregularis* are transcriptionally dependent on the Pi availability. In order to identify the key transcription factor regulating the expression of downstream PHO genes in response to phosphate starvation, we searched for the sequences that correspond to the TFs in PHO pathway of *S. cerevisiae* using the genome sequencing projects of *R. irregularis* DAOM 197198 (Tisserant et al., 2013; Chen et al., 2018), and found a transcription factor called RiPho4. According to the GenBank annotation, *RiPho4* contains 5 exons and 4 introns, a total length of 2052 bp with 1710 bp of ORF (Figure 3A). Using Smart program, it is predicted that *RiPho4* encoding protein has 6 domains, 5 of which are low complex domains (LCDs), while the region from +436 to +515 is a HLH (helix loop helix) domain (Figure 3B), which is one of the specific domains of TFs (Ferré-D'Amaré et al., 1993; Kiribuchi et al., 2004; Carretero-Paulet et al., 2010; Zhu and Huq, 2011). Further the three-dimensional conformation of RiPho4 (Figure 3C) showed that RiPho4 is a typical HLH domain containing protein. Therefore, it is predicted that RiPho4 may serve as a key transcription factor containing a HLH domain in *R. irregularis*.

### RiPho4 is conserved across fungi species

To determine the evolutionary relationships of Pho4 proteins between *R. irregularis* and other different fungi species, we performed the phylogenetic analysis and conserved motif identification. The result shows that RiPho4 is related to AM fungal TFs GmPho4, GcPho4, FgPho4 and FmPho4, and has closely relative to the RdPho4 and RcPho4 from *Rhizophagus diaphanous* and *Rhizophagus clarus* (Figure 3D). RiPho4 protein is >98% identical to Pho4 from *R. diaphanous*, 41% to LtPho4 from the filamentous fungi *Lobosporangium transversale* and 38% to ScPho4 of *S. cerevisiae* which has been reported to be a typical HLH-type transcription factor (Oshima, 1997; Tomar and Sinha, 2014). In addition, we identified RiPho4 functional orthologs with Pho4 from other AMF (Supplementary Figure S2). These *in silico* results suggest that *R. irregularis* RiPho4 is highly conserved across fungi species.

### RiPho4 is induced in mycorrhizal roots

**Figure 4:**
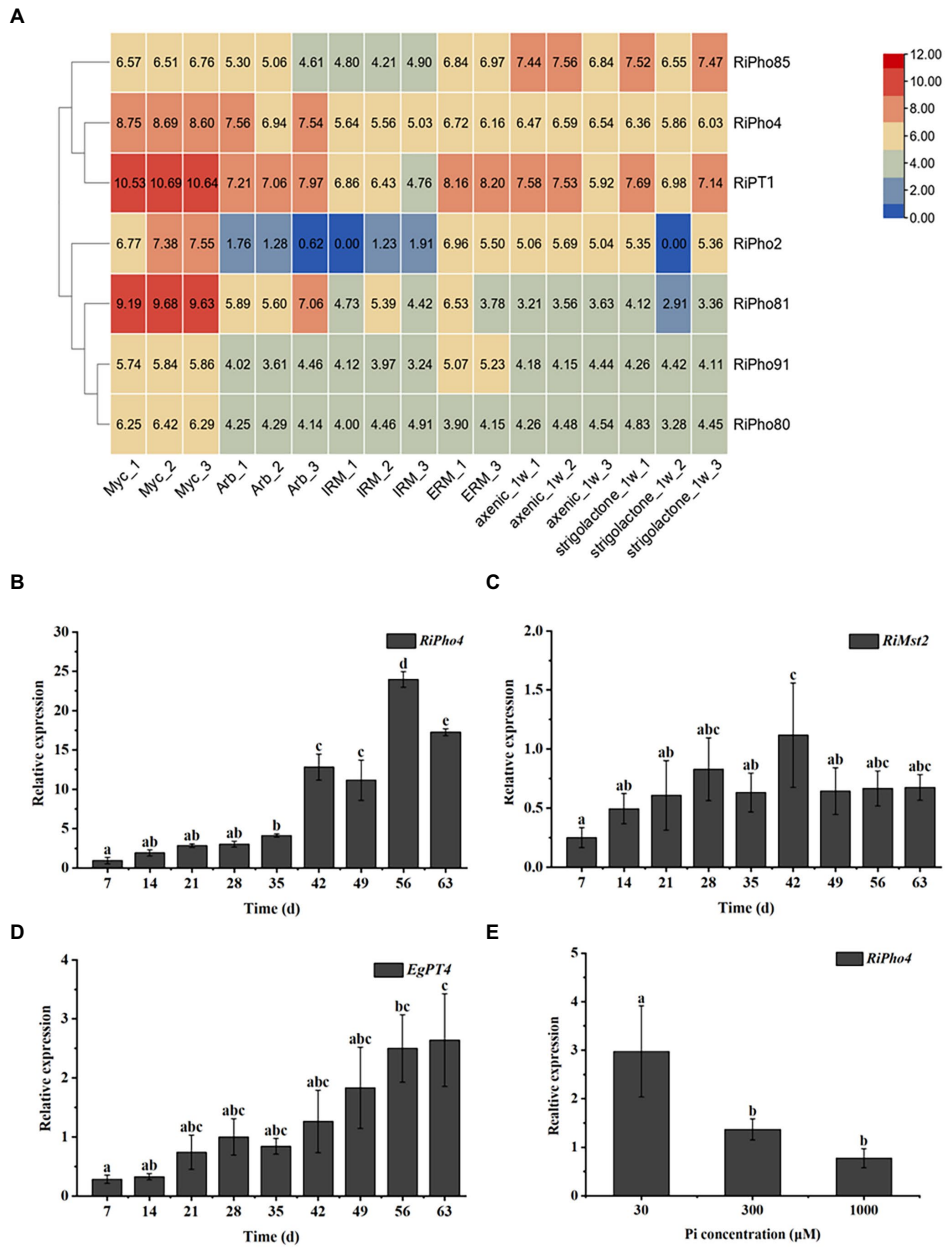
Spatiotemporal expression patterns of *RiPho4* from the AM fungus *R. irregularis*. **(A)** Analysis of the heat diagram for transcriptional levels of *RiPho4* and some PHO pathway genes in different fungal tissues: mycorrhizal roots of *Medicago truncatula* (Myc), arbuscules from *M. truncatula* mycorrhizal roots (Arb), intraradical mycelium (IRM), extraradical mycelium (ERM), axenic germinating spores harvested at a week after inoculation (axenic_1w) and germinating spores at 7 days after GR24 induction (strigolactone_1w). The heatmap of identities was visualized by the TBtools (Chen et al., 2020). **(B–D)** Time-course assay of the expression of *RiPho4*, *RiMst2* and *EgPT4* in *E. grandis* mycorrhizal roots after 7, 14, 21, 28, 35, 42, 49, 56 and 63 days (d) inoculation with *R. irregularis*. *RiEF1α* and *EgUBI3* were used as the reference genes for *R. irregularis* and *E. grandis* gene normalization, respectively. **(E)** Expression analysis of *RiPho4* in mycorrhizal roots of *E. grandis* grown under different phosphate concentrations. The error bars are the means with standard errors of three biological replications. Treatments with the same letters do not differ from others by the Duncan’s test at 5% probability.

To determine the spatiotemporal expression patterns of *RiPho4* from *R. irregularis*, the transcriptional analysis of *RiPho4* and relevant PHO genes were performed in different tissues of *R. irregularis* using the RNA-sequencing data (Zeng et al., 2018, 2020). As shown in Figure 4A, the transcription of *RiPho2, RiPT1, RiPho81, RiPho80,* and *RiPho91* (equally RiPT7, Xie et al., 2022) were up-regulated in mycorrhizal roots when compared with other fungal tissues, while the transcriptional levels of *RiPho4* were induced in both mycorrhizal roots and arbuscules of *R. irregularis*, and *RiPho85* were constitutively expressed in these six different fungal tissues. To confirm the transcriptomic data, we further conducted the qRT-PCR experiments with roots of *E. grandis* colonized with *R. irregularis*. The time-course analysis indicated that *RiPho4* expression was still high in the later stage of mycorrhizal symbiosis, and its transcript reached the highest level at 56 days post inoculation (Figure 4B), this pattern was correlated with the colonization and development processes of *R. irregularis* within *E. grandis* roots (Supplementary Figure S3). Additionally, transcriptional patterns of the monosaccharide transporter gene *RiMst2* and Pi transporter gene *EgPT4*, which are thought to be the AM marker genes (Helber et al., 2011; Che et al., 2022), were also similar to that of *RiPho4* during symbiosis (Figures 4C,D). Therefore, these results indicated that *RiPho4* is induced in the arbuscles of mycorrhizal roots.

### RiPho4 is expressed in response to Pi starvation

To further determine the effect of external Pi concentrations on the transcription of *RiPho4* during the *in planta* phase, *E. grandis* plants inoculated with *R. irregularis* were cultured in the pots supplemented with different phosphate concentrations (30, 300 or 1,000 μM). The qRT-PCR experiment was then performed on the mycorrhizal roots of *E. grandis* subjected to different Pi concentrations as mentioned above. As a result, the expression levels of *RiPho4* were significantly inhibited in roots during high Pi concentrations (300, 1,000 μM) (Figure 4E). This result is consistent with the above findings on PHO gene expression profiles (see Figure 2), suggesting that *RiPho4* is induced during mycorrhizal symbiosis in response to Pi starvation.

### RiPho4 encodes a transcription factor and localizes in nucleus at low phosphate concentrations

**Figure 5:**
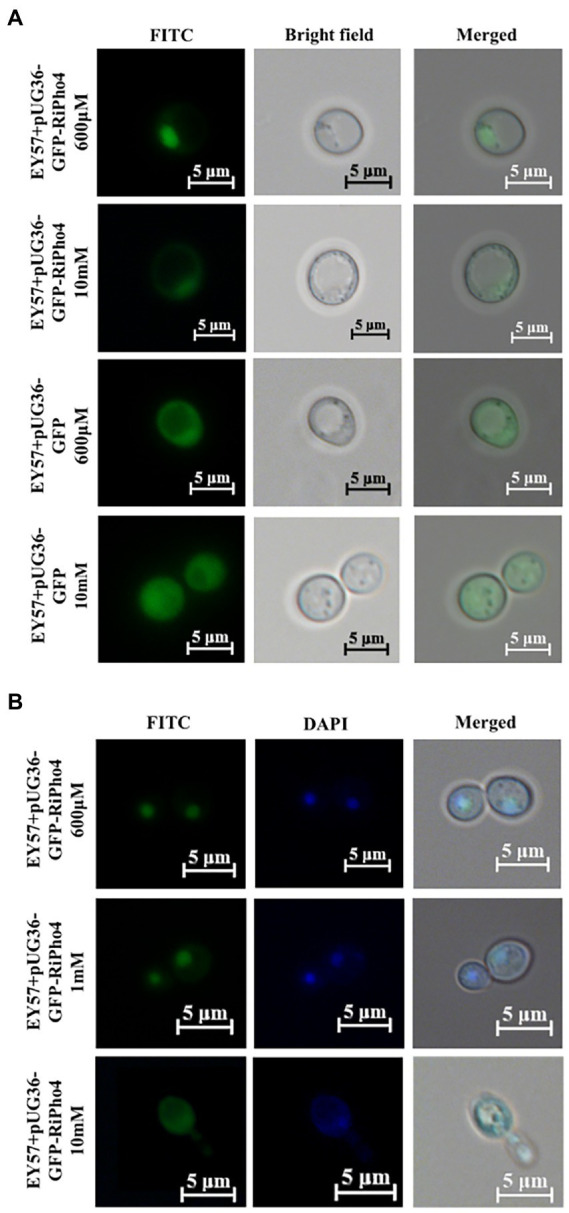
Subcellular localization of RiPho4 in *Saccharomyces cerevisiae* grown under different phosphate conditions. Fluorescence microscope images of *S. cerevisiae* EY57 cells expressing pUG36-GFP or pUG36-GFP-RiPho4. **(A)** Localization of RiPho4 was captured by fluorescence microscopy, the pUG36 containing the GFP reporter gene was used as the control, showing the fluorescence signal in the cytoplasm. The yeast cells were grown in YNB liquid medium with different Pi (600 μM, 10 mM KH_2_PO_4_) treatments for 12 h. **(B)** The transformed cells were grown in YNB medium under different Pi (600 μM, 1 mM, 10 mM K_2_HPO_4_) conditions for 12 h. FITC showed the green fluorescence channel; DAPI presented transformed yeast cells stained with DAPI dye. Scale bars, 5 μm.

Previous studies have found that ScPho4 protein in *S. cerevisiae* imports into the nucleus under low phosphorus supply, whereas the Pho4 exports into the cytoplasm nucleus of yeast cells exposed to high phosphorus conditions (Kaffman et al., 1994; Byrne et al., 2004; Urrialde et al., 2015). To investigate whether RiPho4 has a similar function to ScPho4 from *S. cerevisiae*, we carried out the subcellular localization experiments in yeast cells. As expected, GFP-RiPho4 fusion protein was localized in the nuclei of yeast strain EY57 cells during low Pi treatment (600 μM), while this fusion protein was found in the cytoplasm of yeast cells exposed to high P concentration (10 mM) (Figure 5A). To further verify the localization results as mentioned above, we used a labeling dye DAPI, which can bind to DNA to label the nuclei of yeast cells. The co-localization analysis indicated that the expression of GFP-RiPho4 fusion protein in yeast cells was confirmed to be correctly localized in the nuclei of yeast cells under Pi-limited (600 μM and 1 mM) conditions (Figure 5B). In conclusion, these results reveal that RiPho4 protein locates in the nucleus under low Pi conditions and may serve as a transcription factor in fungal cells.

### RiPho4 is required for arbuscule development

**Figure 6:**
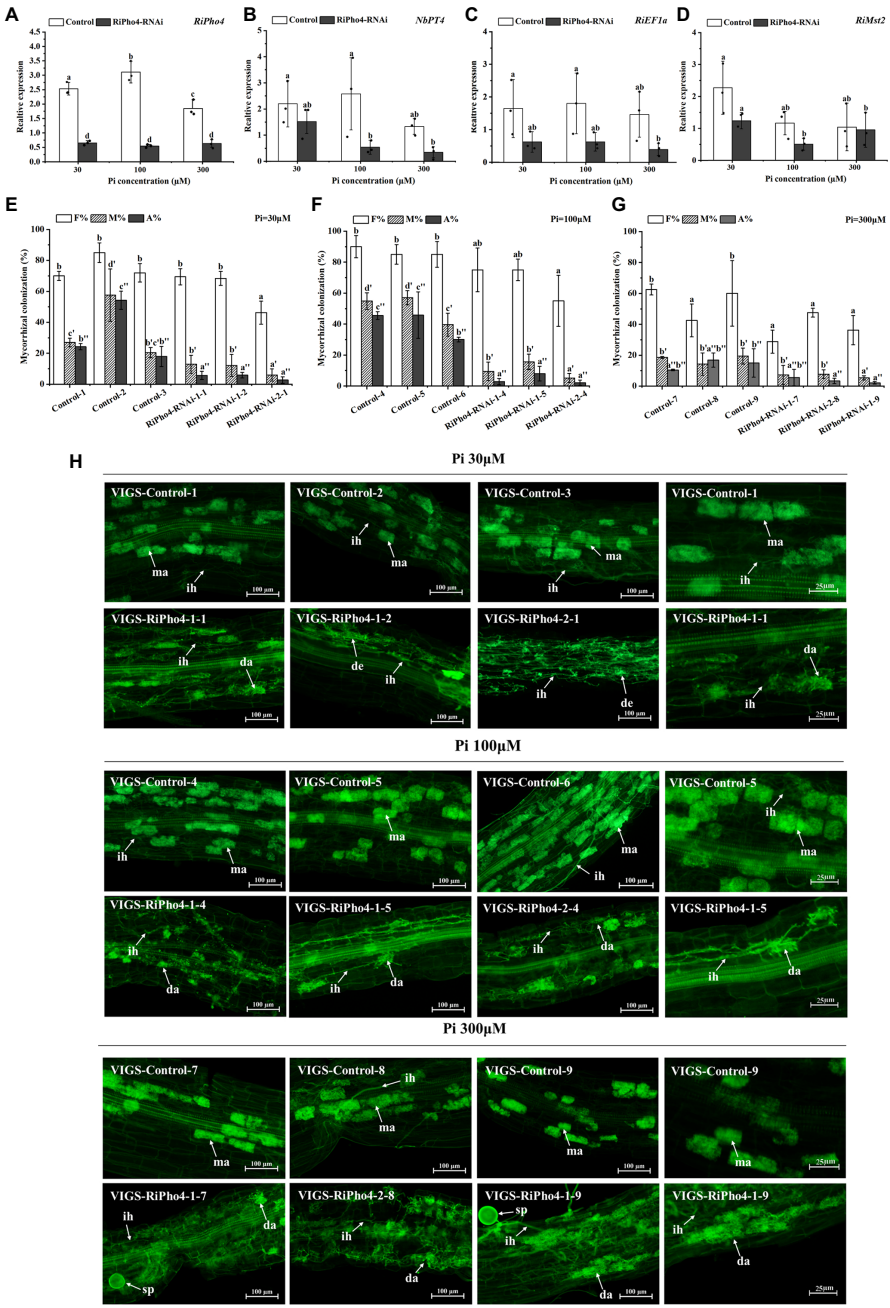
Molecular and arbuscular mycorrhizal phenotypes of virus-induced gene silencing (VIGS) of *RiPho4* in tobacco (*Nicotiana benthamiana*) roots colonized by *R. irregularis* grown under different Pi conditions. **(A–D)** Expression levels of *RiPho4*, *NbPT4*, *RiEF1α* and *RiMst2* in mycorrhizal tobacco *RiPho4-RNAi* roots under different phosphate conditions, estimated by quantitative RT-PCR. *R. irregularis RiEF1a* and *N. benthamiana NbTEF1a* were used as the endogenous genes for normalization of *RiPho4*, *RiMst2*, *NbPT4* and *RiEF1α* expression, respectively. The data represented the means of three biological replicates. Bars indicated the standard errors of means. Means designated with the same letters are not significantly different (*p* ≥ 0.05) according to Duncan’s multiple range test. **(E–G)** Mycorrhizal colonization levels between the control and *RiPho4-RNAi* roots exposed to different Pi (30 μM, 100 μM and 300 μM K_2_HPO_4_) concentrations were determined after the WGA488 staining. F%, the total colonization frequency; M%, the percentage of mycorrhizal intensity; A%, the percentage of arbuscule abundance. **(H)** Fluorescence microscopic images of *R. irregularis* arbuscules in control and *RiPho4-RNAi* roots grown under different Pi conditions. The arrows and letters in the figure represent different structures of *R. irregularis*: ma, mature arbuscules; da, degraded arbuscules; de, dead arbuscules; ih, intraradical hyphae; sp., spores. Scale bars, 100 μm (1–3 columns), 25 μm (the 4th column).

In order to further study the roles of *RiPho4* in AM symbiosis, we knocked down *RiPho4* expression by the VIGS method. We designed two RNAi regions to target the corresponding specific regions of *RiPho4* that was very divergent from the other genes containing bHLH domains in *R. irregularis* to avoid off-target silencing (see Supplementary Figure S5). First, we detected the transcriptional levels of *RiPho4* in mycorrhizal roots of *N. benthamiana* under three different Pi concentrations (30, 100, or 300 μM). The results showed that the transcriptional levels of *RiPho4* in the *VIGS-RiPho4* roots were significantly reduced by more than 60%, when compared with the control roots, indicating that expression of *RiPho4* was effectively knocked down (Figure 6A). Expressions of the monosaccharide transporter gene *RiMst2*, AM fungal reference gene *RiEF1α* and Pi transporter gene of *N. benthamiana NbPT4*, which are considered as symbiotic marker genes (Helber et al., 2011; Kikuchi et al., 2016; Xie et al., 2022), were significantly decreased in *RiPho4-RNAi* roots under different Pi concentrations (Figures 6B–D).

Further observation of the mycorrhizal colonization uncovered the distinguishable AM phenotype between the *RiPho4-RNAi* and control roots during AM symbiosis under different Pi concentrations. Relative to the controls, the total AMF colonization in most of *RiPho4-RNAi* roots showed a slightly but not significantly decrease, while silencing of *RiPho4* obviously decreased the mycorrhizal intensity and arbuscule abundance in roots (Figures 6E–G). Moreover, more arbuscules in the *RiPho4-RNAi* roots were abnormal, or degenerating under different Pi conditions (Figure 6H). Collectively, these findings reveal that *RiPho4* is essential for arbuscule development.

### RiPho4 plays an important role in regulating the symbiotic Pi absorption during AM symbiosis

**Figure 7:**
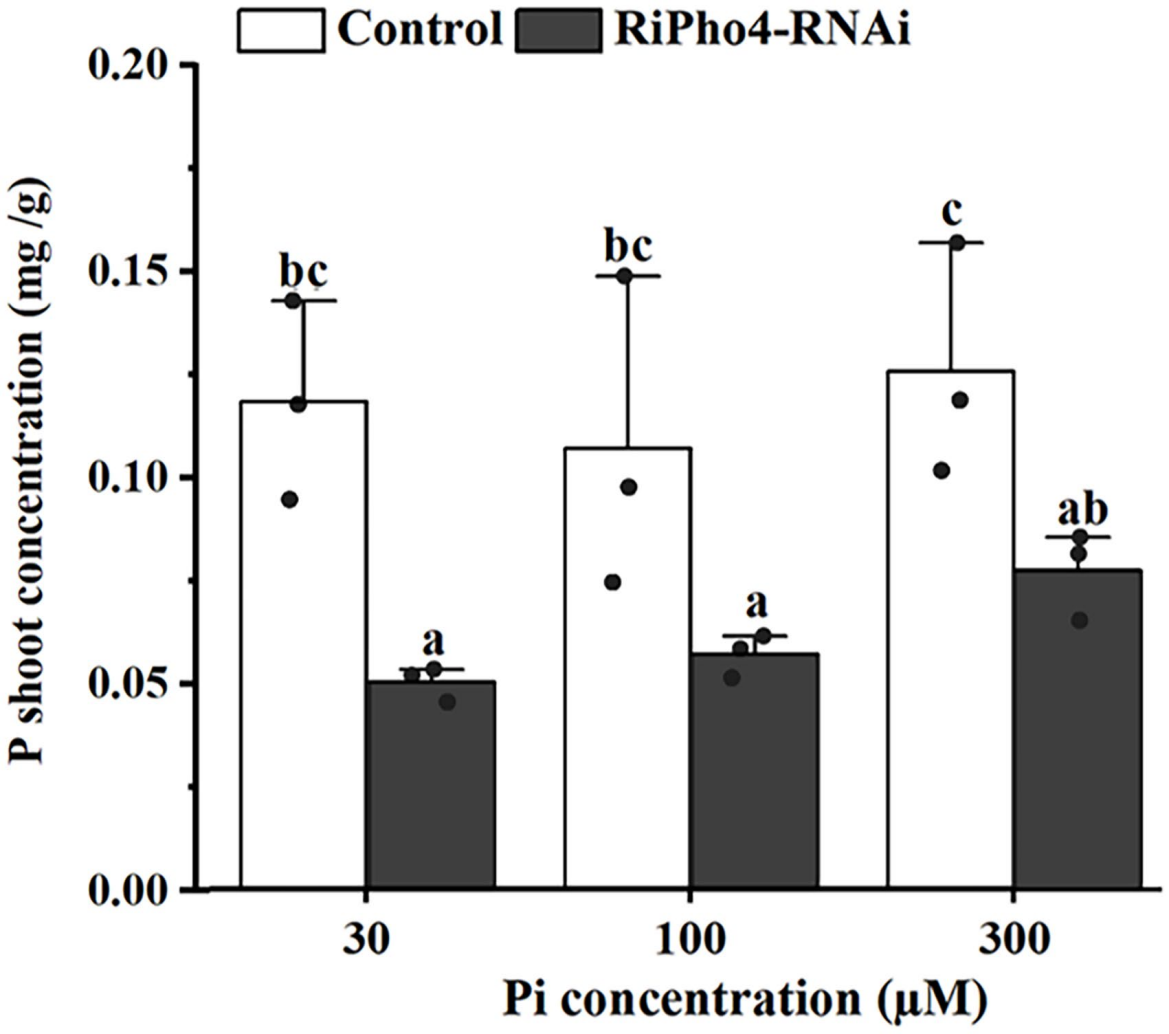
Phosphate concentrations in the tobacco (*N. benthamiana*) shoots of the control and VIGS of *RiPho4* treatments under different Pi conditions. The data represented the means with SE. Different letters indicated the significant differences which statistically analyzed by Duncan’s multiple range test at the alpha = 0.05 level.

To determine whether RiPho4 regulates the symbiotic Pi transport in AM symbiosis of *N. benthamiana*, we examined the total P concentration of AM tobacco shoots of control and *RiPho4-RNAi* plants grown under three different Pi (30, 100, or 300 μM) conditions. As a result, the P concentration in AM plants of RiPho4-silenced lines were significantly reduced compared with control plants (Figure 7). This result indicated that RiPho4 may regulate Pi transport at the symbiotic interface of arbuscular mycorrhizas.

### Knock-down of RiPho4 affects the expression of PHO pathway genes in *Rhizophagus irregularis* during AM symbiosis

**Figure 8:**
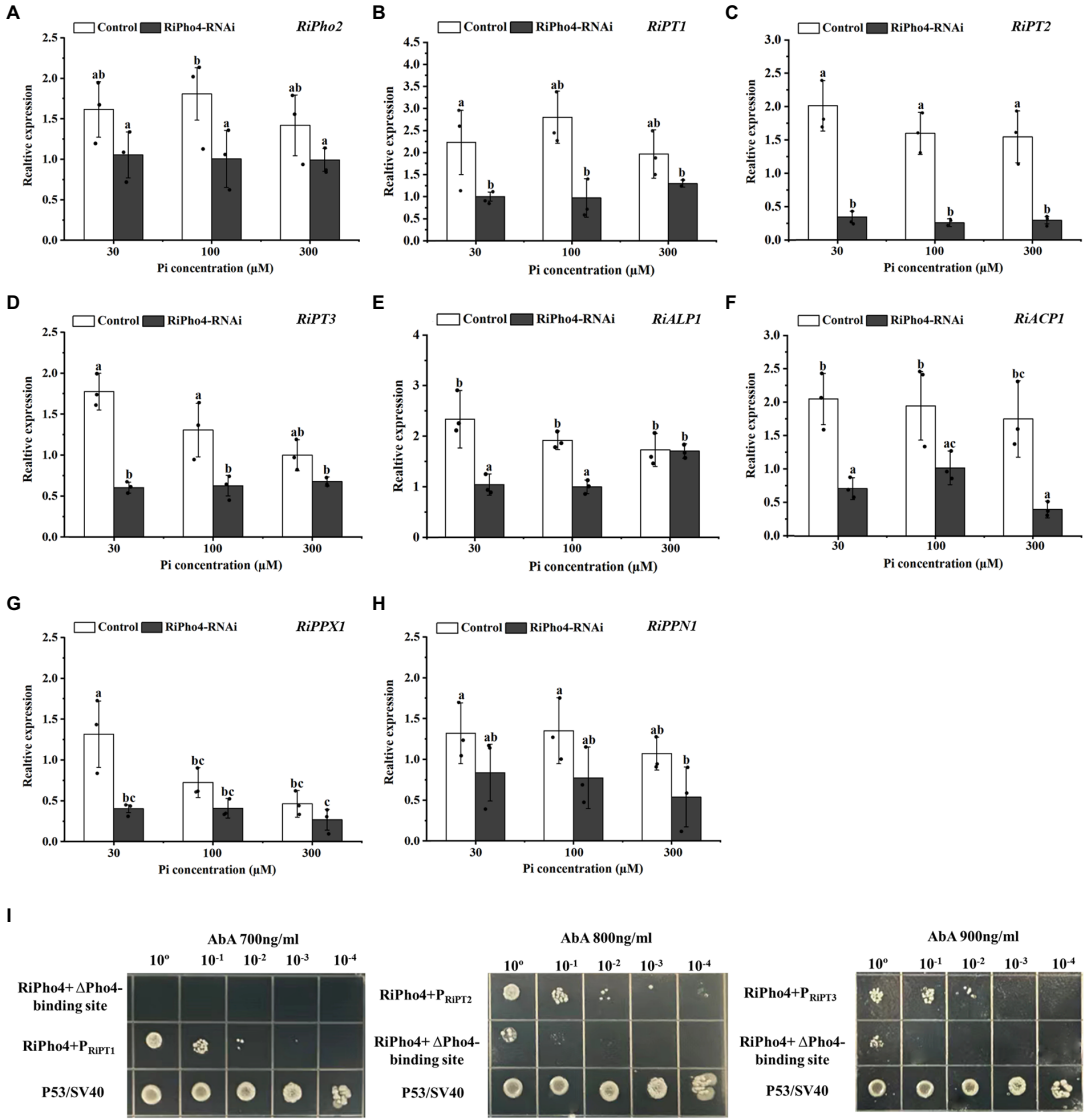
RiPho4 regulates the expression of downstream genes involved in Pi transport and metabolism processes in *R. irregularis*. **(A–H)** Expression analysis of *R. irregularis* genes engaged in PHO pathway between *RiPho4-RNAi* and control roots under different phosphate concentrations. Expression levels of *RiPho2* **(A)**, Pi transport genes *RiPT1, RiPT2, RiPT3* **(B–D)**, phosphatase related genes *RiALP1, RiACP1* (E-F), *RiPPX1* and *RiPPN1* related to Poly-P metabolism **(G,H)** in response to different phosphate concentrations were estimated by real-time qRT-PCR analysis. Different letters indicated significant differences between control and RNAi lines. The differences between samples were analyzed using Duncan’s new multi-range test at the alpha = 0.05 level. **(I)** Yeast one-hybrid analysis of the interaction between RiPho4 with promoters of *RiPT1*, *RiPT2*, and *RiPT3*. The promoter regions of *RiPT1*, *RiPT2* and *RiPT3* containing the *cis*-acting elements CACGTG/T named P_RiPT1_, P_RiPT2_, and P_RiPT3_. The identification of interaction between RiPho4 and the promoter of *RiPT1*, *RiPT2*, or *RiPT3* under the screened even higher AbA inhibition concentration indicated in Fig. S4. Yeast cells carrying both pGBKT7-P53 and pGADT7-SV40 grown on SD/−Leu were used as the positive control, P_RiPT1,_ P_RiPT2,_ P_RiPT3_-ΔPho4-binding sites with pGADT7-RiPho4 were used as the negative controls. ΔPho4-binding site indicates the deletion of the Pho4 binding site (CACGTG/T) elements located in P_RiPT1,_ P_RiPT2,_ P_RiPT3_ promoters. 10-fold serial dilutions of yeast cells were spotted on plates, and the initial yeast concentration was OD_600_ = 0.2.

To confirm the function of RiPho4 in the PHO pathway of *R. irregularis*, qRT-PCR was used to explore the effect of *RiPho4* knock-down on the PHO pathway genes in mycorrhizal *N. benthamiana* roots exposed to different Pi concentrations. The results showed that *RiPho2, RiPT1*, *RiPT2* and *RiPT3* were significantly decreased in RiPho4-silenced roots under low Pi conditions (30–100 μM) when compared with control roots (Figures 8A–D). Furthermore, the expression levels of genes involved in Pi and Poly-P metabolism in *R. irregularis*, such as *RiALP1*, *RiACP1, RiPPX1* and *RiPPN1* (Xie et al., 2022), were also inhibited in the *RiPho4-RNAi* roots when compared to the controls (Figures 8E–H), suggesting that loss of *RiPho4* function results in a reduction of Pi and Poly-P metabolisms under Pi-limited conditions. Therefore, these results indicate that *RiPho4* may positively regulate the transcriptional levels of the downstream genes of PHO pathway in *R. irregularis* during AM symbiosis under Pi-deficient conditions.

### RiPho4 directly regulates the Pi transporter genes of PHO pathway from *Rhizophagus irregularis*

To confirm that *RiPho4* encodes a transcription factor to regulate PHO genes, we tested its ability to interact with Pi transporters of *R. irregularis* by the yeast one-hybrid (Y1H) assay. It is known that the common binding motif of Pho4 transcription factor is CACGTG/T (Secco et al., 2012; Tomar and Sinha, 2014), we therefore selected the same CREs (*cis*-regulatory elements) in the promoters of three Pi transporter genes *RiPT1, RiPT2,* and *RiPT3* for further studies (see Supplementary Table S5).

As a result, the Y1HGold yeast cells containing pAbAi vector with P_RiPT1_, P_RiPT2_, or P_RiPT3_ were significantly inhibited at 400, 600, and 800 ng/ml AbA, respectively (see Supplementary Figure S4). In the Y1H assay, the yeast cells containing AD-RiPho4 plasmid and wild-type promoter P_RiPT1_, P_RiPT2_, or P_RiPT3_ still grew well on medium supplemented with 700 ng/ml, 800 ng/ml, or 900 ng/ml AbA, while the yeasts containing mutant promoters without Pho4-binding sites (CACGTG/T) were strongly inhibited under such conditions (Figure 8I). These data showed that RiPho4 protein interacts with the promoters of *RiPT1*, *RiPT2*, and *RiPT3* through the Pho4-binding sites (CACGTG/T). Therefore, the transcription factor RiPho4 can directly regulate the Pi transporter genes *RiPT1*, *RiPT2* and *RiPT3* in PHO pathway. Collectively, the Y1H results implicate that RiPho4 may act as a transcriptional activator in the PHO pathway in *R. irregularis*.

## Discussion

Recent years, more researchers have reported the physiological responses of Eucalyptus species to low Pi stress (Wu et al., 2014; Niu et al., 2015; Bahar et al., 2018), and focused on the effects of different P levels on plant biomass and P content (Xu et al., 2001; Standish et al., 2007; Bichara et al., 2021), but little study on the molecular mechanisms of the interaction between AMF and Eucalyptus plants. Although the PHO pathway responsible for Pi absorption, transport and metabolism has been described in several fungi species (Kerwin and Wykoff, 2009; Zheng et al., 2020; Ahmed et al., 2022), the regulatory mechanisms of Pi nutrient exchange between AMF and host plants through PHO pathway is still partially understood so far (Xie et al., 2016, 2022). In this study, we focus on the AM fungus *R. irregularis* mediating Pi uptake and homeostasis in AM symbiosis of *E. grandis* by investigating the expression, localization and function of RiPho4, a transcription factor of the PHO pathway.

### The PHO pathway of AM fungus plays a Key role in phosphate absorption during AM symbiosis

It has been reported that AMF can significantly promote Pi uptake and Pi stress adaptation abilities of host plants in both field and laboratory conditions (Abdel-Fattah, 2001; Kobae, 2019; Wang et al., 2019). Accordingly, a large number of studies have shown that the growth indices of AM plants, such as plant height, stem diameter, leaf area, root volume, shoot, root dry weight and P content, were significantly higher than those of NM plants under phosphorus limitation (Pumplin and Harrison, 2009; Frosi et al., 2016; Tian et al., 2017; Carballar-Hernandez et al., 2018; Wang et al., 2019). Especially at low Pi concentration, the host plants have high dependence to AMF (Chu et al., 2013). The results and data of the overall growth and physiological status of *R. irregularis*-*E. grandis* interaction in this study (Supplementary Figure S1; Figures 1A–E) are consistent with the previous results. AMF are sensitive to P supply and a low to moderate supply is required (Ezawa et al., 2002), the intensities of fungal ALP, ACP and SDH activities reflecting the metabolic activity and function of AMF also decreased with the increasing P input (Hamel et al., 1990; Saito, 1995; Vivas et al., 2003; Li et al., 2017; Wang et al., 2017). Similarly, high Pi supply strongly suppressed the AMF colonization and arbuscule formation (see Figure 1F) as well as the intensities of AM fungal ALP, ACP, and SDH activities (see Figures 1G–I; Supplementary Table S1) in this study. Because ACP and ALP are involved in the hydrolysis of Poly-P in the AMF (Ezawa et al., 2002), it can be considered that AMF greatly improved P nutrient of host plants by Poly-P hydrolysis in the IRM and apoplast when phosphate concentration was limited. The previous studies have found that the Pi uptake of mycorrhizal plants includes the direct uptake pathway and mycorrhizal uptake pathway (Smith and Smith, 2011). When Pi concentration was limited, Pi absorption of symbiotic plants is mainly through the mycorrhizal pathway (Zhang et al., 2021). However, when the phosphorus supply is sufficient, the direct pathway is activated within host plants to uptake from root surface, and the AMF has few Pi contribution (Chu et al., 2020). This is consistent with the results that the AMF colonization decreased and the phosphatase activities were decreased in mycorrhizal roots when Pi concentration was high (see Figure 1). In conclusion, plants absorb Pi mainly *via* the mycorrhizal pathway under low P environments, and AMF plays an important role in the growth of *E. grandis*.

Until now, molecular mechanisms by which AMF regulate Pi efflux from the IRM to symbiotic interface are partially understood (Wang et al., 2017; Nguyen and Saito, 2021; Xie et al., 2022). In yeast, several studies have implicated that transcription of PHO pathway genes are closely related to the Poly-P metabolism and cytosolic Pi transportation (Vardi et al., 2014; Desfougeres et al., 2016). The PHO pathway has been extensively characterized in yeast but less in AMF (Tisserant et al., 2012; Xie et al., 2016, 2022). In the case of yeasts and AMF, the homeobox transcription factor Pho2, Pi transporter genes, VTC complex VTC1/2/4, cyclin Pho80, CDK inhibitor Pho81, Cyclin-dependent kinase Pho85 are all controlled in the PHO pathway and in response to Pi deficiency (Lenburg and O’Shea, 1996; Ezawa et al., 2002; Tomar and Sinha, 2014; Xie et al., 2022). Similar identification was conducted on these homologous genes in *Neurospora crassa* (Gras et al., 2013). Through qRT-PCR analysis, it was found that the PHO pathway genes in *R. irregularis* were generally more active at low Pi levels than at medium and high Pi levels (see Figure 2). Besides, the transcriptional levels of *RiALP1* and *RiACP1* were influenced by high Pi supply (see Figures 2F,G) were consistent with the ALP and ACP staining results (see Figures 1G–I; Supplementary Table S1). Therefore, it is predicted that the PHO pathway genes of *R. irregularis* may play important roles in promoting plant Pi absorption during AM symbiosis under low Pi conditions, and the regulon which control the transcription of PHO pathway genes during Pi starvation is worthy to be further investigated.

### RiPho4 acts as a key transcription factor of the PHO pathway in *Rhizophagus irregularis*

Very recently, it has been found that the core components of PHO pathway are evolutionarily conserved among AMF and yeast species (Zhou et al., 2021). In *S. cerevisiae*, Pho4 is known as a helical loop–helix (HLH) transcription factor to activate the expression of PHO downstream genes in response to Pi limitation (Vogel et al., 1989; Komeili and O’Shea, 1999; Urrialde et al., 2015). ScPho4 is a Pi-sensitive core regulation factor (Tomar and Sinha, 2014). Here, RiPho4, the homologous protein of ScPho4 in *R. irregularis*, is highly conserved in fungi species containing the HLH domain to bind to DNA (see Figure 3; Supplementary Figure S2). Therefore, RiPho4 can be considered as an important transcription factor of the PHO pathway in *R. irregularis*.

From expression patterns of *RiPho4* (see Figure 4), it is more active in mycorrhizal roots and arbuscules during symbiosis, indicating that RiPho4 may function in the Pi nutrient exchange at the symbiotic interface, especially in arbuscules (Smith and Smith, 1997; Karandashov et al., 2004; Harrison, 2012; Luginbuehl and Oldroyd, 2017). Moreover, the transcription of *RiPho4* is dependent on the Pi availability, this is similar to *Pho4* in filamentous fungi (Peleg et al., 1996; Gras et al., 2013; Tomar and Sinha, 2014). The RiPho4 protein location in the nuclei of yeast cells is also dependent on Pi availability. When facing to the high phosphate concentrations, RiPho4 can be moved to the cytoplasm (see Figure 5). This finding is similar to Pho4 location patterns of yeasts and filamentous fungi (Peleg et al., 1996; Byrne et al., 2004; Urrialde et al., 2015). It is well-known that one of the mechanisms regulating the activation of TFs is cytoplasmic retention and subsequent translocation into the nucleus due to external stimuli (Reich and Liu, 2006; Hao and O’Shea, 2012). And for RiPho4, the transcription factor in the cytoplasm subsequently translocates into the nucleus in response to low Pi stimulus, like ScPho4. Therefore, our findings reveal that the transcription factor RiPho4 in *R. irregularis* is induced during P starvation, and may play a key role in the regulation of Pi uptake and homeostasis during AM symbiosis.

### RiPho4 regulates arbuscule development and Pi concentration of mycorrhizal plants through modulating the PHO genes in *Rhizophagus irregularis*

Up to date, few Pi transporter genes of PHO pathway in AMF, such as *GigmPT* and *RiPT7*, have been functionally described by gene silencing, and knock-down of *GigmPT* or *RiPT7* leads to fungal growth arrest and impaired arbuscule development (Xie et al., 2016, 2022). Correspondingly, in our study, *RiPho4* silencing also results in the obvious phenotype of arbuscule degradation (see Figure 6). The previous studies have shown that the process of AMF Pi delivery to plant cells serve as a signal to maintain the arbuscule development (Javot et al., 2007; Xie et al., 2016, 2022). However, Pi levels of mycorrhizal tobacco were significantly reduced in the *RiPho4-RNAi* lines when compared with the control lines (see Figure 7), suggesting that the loss of *RiPho4* function results in the hinder of Pi transportation from the arbuscules to host plants. Therefore, RiPho4 functions in maintaining the arbuscule development, resulting from its role in promoting Pi exchange at the symbiotic interface of mycorrhizas. Indeed, this regulatory roles of fungal Pho4 proteins in Pi uptake and homeostasis have been demonstrated. Previous studies showed that *NUC-1* (*Pho4* homologous gene) from *N. crassa* is considered to be a factor to activate the transcription of Pi transporter genes (Kang and Metzenberg, 1990; Gras et al., 2013), and in AMF, Pho4 is predicted to have a regulatory role on the PHO responsive genes (Xie et al., 2016; Zhou et al., 2021). As expected, in *R. irregularis*, the knock-down of *RiPho4* by VIGS resulted in the significant decrease in expression levels of Pi transporter genes, Pi and Poly-P metabolism genes under Pi limited conditions (see Figures 8A–H). From this result, it is suggested that RiPho4 regulates Pi transport and homeostasis at the symbiotic interface through controlling the PHO gene expression.

Next, question is how RiPho4 regulates the transcription of PHO genes in *R. irregularis*. We used the Y1H assay to preliminarily address this issue. As shown in Figure 8I, the RiPho4 protein interacted with the promoters of Pi transporter genes *RiPT1*, *RiPT2* and *RiPT3* in *R. irregularis* through binding to the CACGTG/T sites. On the basis of the results, it is concluded that RiPho4 is able to directly regulate Pi transporter genes of PHO pathway. Since other downstream genes of PHO pathway, such as *RiVTC1, RiVTC2* and *RiALP1*, also contain the Pho4-binding sites (CACGTG/T), it is predicted that RiPho4 may also have regulatory functions on these Pi and Poly-P metabolism genes (see Supplementary Table S5). In filamentous fungi, Pi responsive genes containing the Pho4-binding sites, including phosphate permeases and repressible alkaline phosphatase genes, help the cell to survive in the prevailing low Pi environment (Lenburg and O’Shea, 1996; Gras et al., 2007; Leal et al., 2007; Tomar and Sinha, 2014). These evidences indicate that RiPho4 can directly regulate the downstream genes of PHO pathway to control Pi uptake and homeostasis during AM symbiosis. However, whether RiPho4 binds to a large number of Pi responsive genes involved in the PHO pathway of *R. irregularis* need to be further identified in future. Taken together, it is proposed that RiPho4 as a transcription factor regulates arbuscule development and symbiotic Pi homeostasis through controlling the downstream genes in the PHO Pathway of *R. irregularis*.

### A model of the key regulon RiPho4, which functions in the control of PHO genes in AM fungus during symbiosis

**Figure 9:**
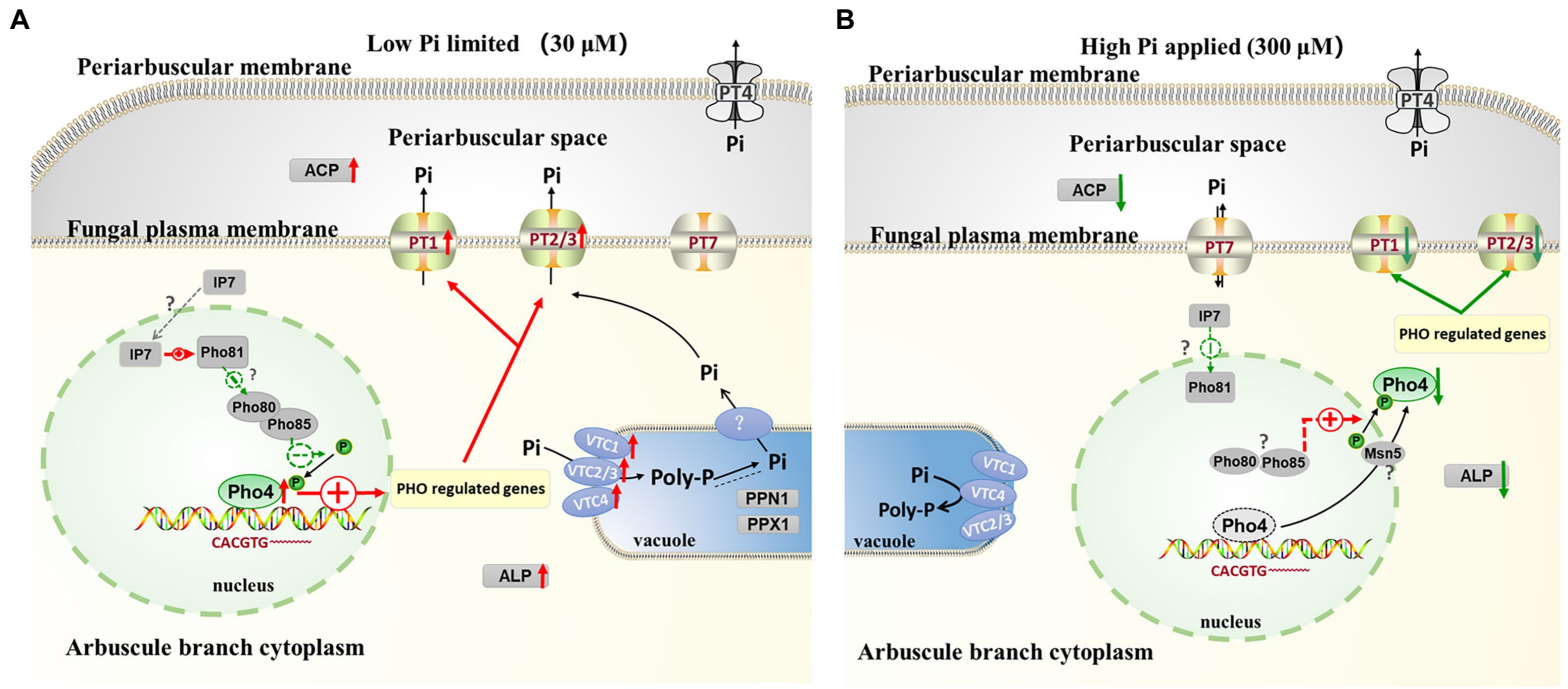
Schematic representation of the function of RiPho4 protein regulating phosphate transport and homeostasis in *R. irregularis* during AM symbiosis. **(A)** Under Pi limiting conditions, it is predicted that RiPho4 may be hypo-phosphorylated by RiPho80-RiPho85 complex (Jeffery et al., 2001), and Pho4 locates in nucleus to activate the transcription of downstream genes involved in the PHO pathway, such as *PT1/2/3*, *ACP*, *ALP*, *PPN1* and *PPX1*. **(B)** Under high Pi conditions, the SPX domain-containing Pi transporter RiPT7 is responsible for Pi transport at the symbiotic interface (Xie et al., 2022), whereas the IP7 signal is predicted to be inhibited and the Pho85-Pho80 complex may phosphorylate Pho4 protein (Lee et al., 2008), which is exported from nucleus to cytoplasm, then switches off the PHO pathway. Negative and positive regulatory effects were indicated by green and red arrows, respectively. The relationships between the well-known genes and those not yet determined were shown by the solid and dashed heads, respectively. IP7, inositol heptakisphosphate; Poly-P, polyphosphate; Pi, inorganic phosphate. The green up and red down arrows next to genes indicated that transcript levels of genes were increased and decreased, respectively.

According to our results and previous studies (Oshima, 1997; Tomar and Sinha, 2014; Zhou et al., 2016; Ahmed et al., 2022), we proposed a working model in which RiPho4 acts as a transcription factor controlling the PHO genes to regulate Pi transport and homeostasis at the symbiotic interface (Figure 9). In this model, under low Pi conditions (Figure 9A), in arbuscules, the level of inositol heptakisphosphate (IP7), an evolutionary conserved metabolite (Lee et al., 2007), may increase. This induces the expression increasement of CDK inhibitor RiPho81 function (Lee et al., 2008; York and Lew, 2008), thereby preventing the formation of RiPho85 and RiPho80 complex (Schneider et al., 1994; Huang et al., 2007; Lee et al., 2008). The non-formation of the RiPho85 and RiPho80 complex results in hypo-phosphorylate of RiPho4, which is accumulated in the nucleus and activated with cofactor RiPho2 (Urrialde et al., 2015). Subsequently, RiPho4 binds to the CACGTG/T sites to activate the transcriptions of PHO pathway genes in arbuscules, such as the Pi transporter genes *RiPT1/2/3* and Pi metabolism genes *RiALP1* and *RiACP1*. Moreover, *RiPPN1* and *RiPPX1* are also induced to function in the Poly-P metabolism in vacuoles (Xie et al., 2016; Ezawa and Saito, 2018; Zhou et al., 2021). Therefore, during Pi deficiency, RiPho4 regulates the PHO-related genes to enhance the hydrolyzation of Poly-P in arbuscules, and these free Pi are transported into the PAS *via* the Pi transporters (Xie et al., 2022), then the PAM-located PT4 carriers transport Pi to the plant cells (Harrison et al., 2002; Javot et al., 2007; Che et al., 2022). Conversely, under Pi-sufficient conditions (Figure 9B), the IP7 may be lacking in arbuscules and prevent RiPho81 from inhibiting the kinase activity of RiPho85-RiPho80, thus enabling the phosphorylation of RiPho4 (Jeffery et al., 2001; Huang et al., 2007). However, the phosphorylated RiPho4 is exported from the nucleus into the cytoplasm where it cannot activate the transcription of PHO pathway genes (Oshima, 1997; Kaffman et al., 1998; Ghillebert et al., 2011). Under such conditions, the SPX domain-containing transporter RiPT7 can export Pi into the PAS, and AM-specific PT4 can acquire Pi from PAS (Tomar and Sinha, 2014; Che et al., 2022; Xie et al., 2022).

## Conclusion

In conclusion, this study presented the expression, localization and functions of *RiPho4* from *R. irregularis*. RiPho4 is a transcription factor containing a HLH domain, and is located in the nucleus of yeast cells under low Pi conditions. Further studies revealed that RiPho4 is a key regulatory factor in AM fungus to maintain arbuscule development through regulating the expression levels of the PHO pathway downstream genes in order to handle Pi transport and homeostasis at the symbiotic interface. Our findings provide new insights into the underlying mechanisms by which AMF control phosphate uptake and homeostasis during symbiosis.

## Data availability statement

The original contributions presented in the study are included in the article/Supplementary material, further inquiries can be directed to the corresponding author/s.

## Author contributions

XX and MT designed the experiments and managed the projects. SZ and XF performed the experiments. YN and WW performed data analysis. XF and XX provided advice and guidance on the idea of bioinformatics analysis. SZ, XX, and MT wrote the manuscript. HC assisted with the interpretation of the results. All authors contributed to the manuscript read, edited, and approved the current version.

## Funding

This research was funded by the National Natural Science Foundation of China (32170116 and 32071639), the Key Projects of Guangzhou of Science and Technology Plan (grant no. 201904020022), and the Laboratory of Lingnan Modern Agriculture Project (NZ2021025), the Guangdong Basic and Applied Basic Research Foundation (grant no. 2022A1515012013).

## Conflict of interest

The authors declare that the research was conducted in the absence of any commercial or financial relationships that could be construed as a potential conflict of interest.

## Publisher’s note

All claims expressed in this article are solely those of the authors and do not necessarily represent those of their affiliated organizations, or those of the publisher, the editors and the reviewers. Any product that may be evaluated in this article, or claim that may be made by its manufacturer, is not guaranteed or endorsed by the publisher.

## Footnotes

^1^ http://blast.ncbi.nlm.nih.gov/Blast.cgi

^2^ http://smart.embl.de/

^3^ https://swissmodel.expasy.org/

^4^ https://meme-suite.org/meme/tools/meme

^5^ http://bioinformatics.psb.ugent.be/webtools/plantcare/html/
