# Supplementary material for: A transcriptional activator from Rhizophagus irregularis regulates phosphate uptake and homeostasis in AM symbiosis during phosphorous starvation
Source: Front Microbiol. 2023 Jan 20;13:1114089. doi: 10.3389/fmicb.2022.1114089 (PMC9895418; doi:10.3389/fmicb.2022.1114089)
Supplement: Supplementary file 1 [file Data_Sheet_1.docx]

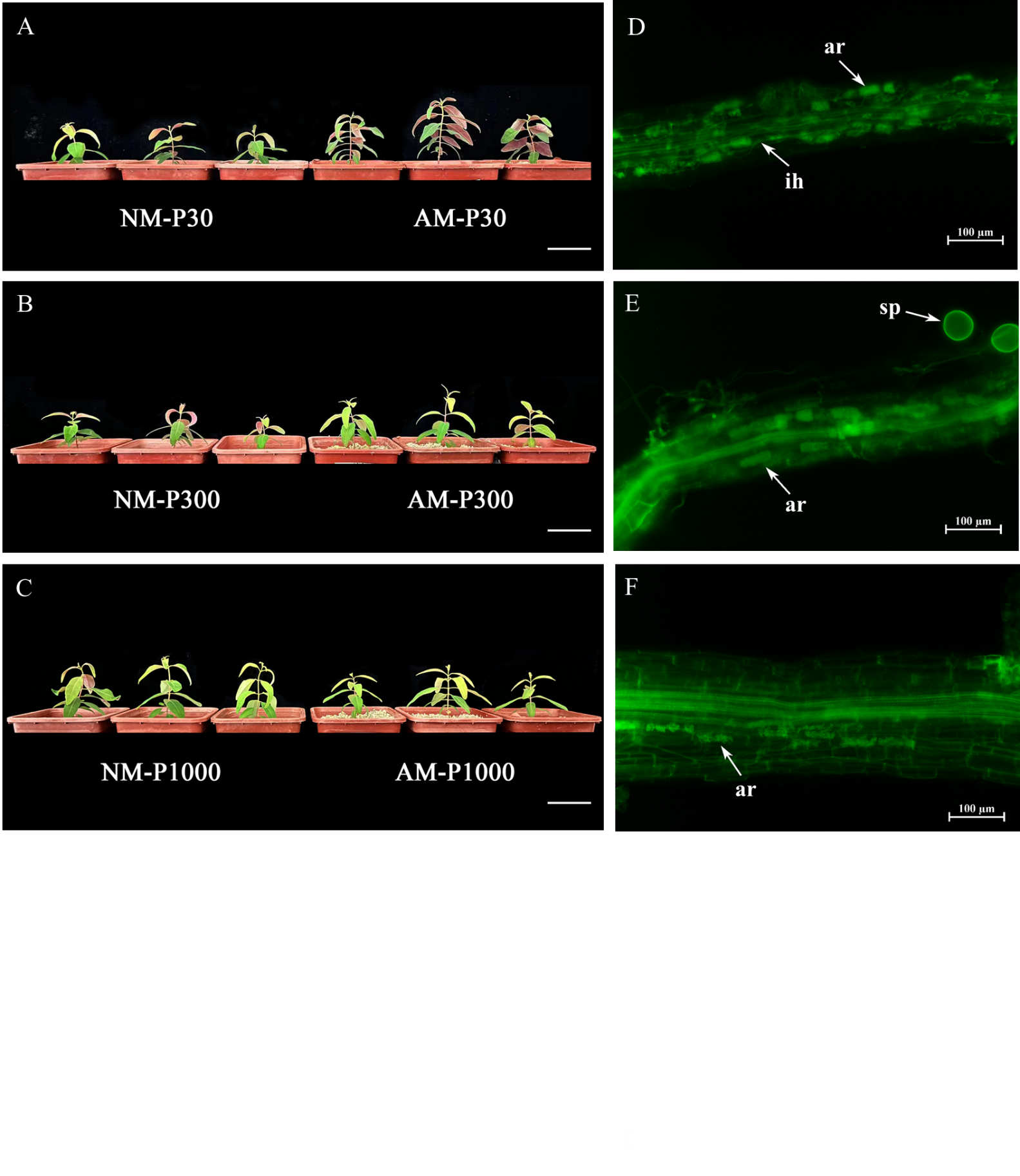


**Fig. S1. Overall growth of *Eucalyptus grandis* with or without AM fungus *Rhizophagus irregularis* at different phosphate concentrations.** (A-C) The phenotype of mycorrhizal *E. grandis* roots at 100, 300 and 1000 μM phosphate concentrations, respectively. Scales bars in the left panels represent 5 cm. (D-F) The one-to-one mapping pictures right represent arbuscular mycorrhizal phenotypes of *E. grandis* in the corresponding phosphate concentration. In figures, ih denotes intraradical hyphae, ar denotes arbuscule and sp denotes spores. Scale bars in right channels represent 100 µm.


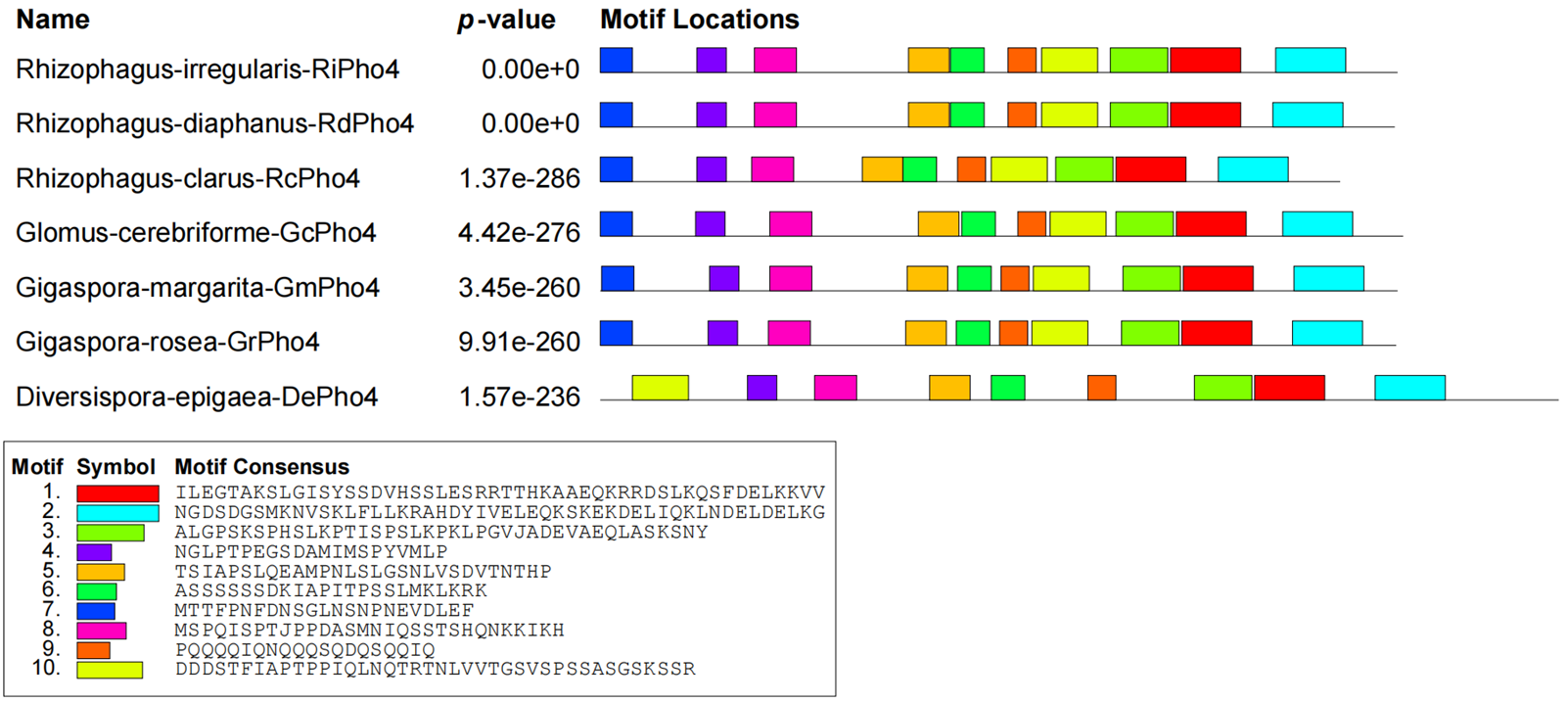


**Fig. S2. Conservative analysis of RiPho4 and its homologous proteins from other AM fungi.** The conserved regions of proteins were analyzed by the Meme (https://meme-suite.org/meme/tools/meme), and the meaning of different colored squares referred to the legend.


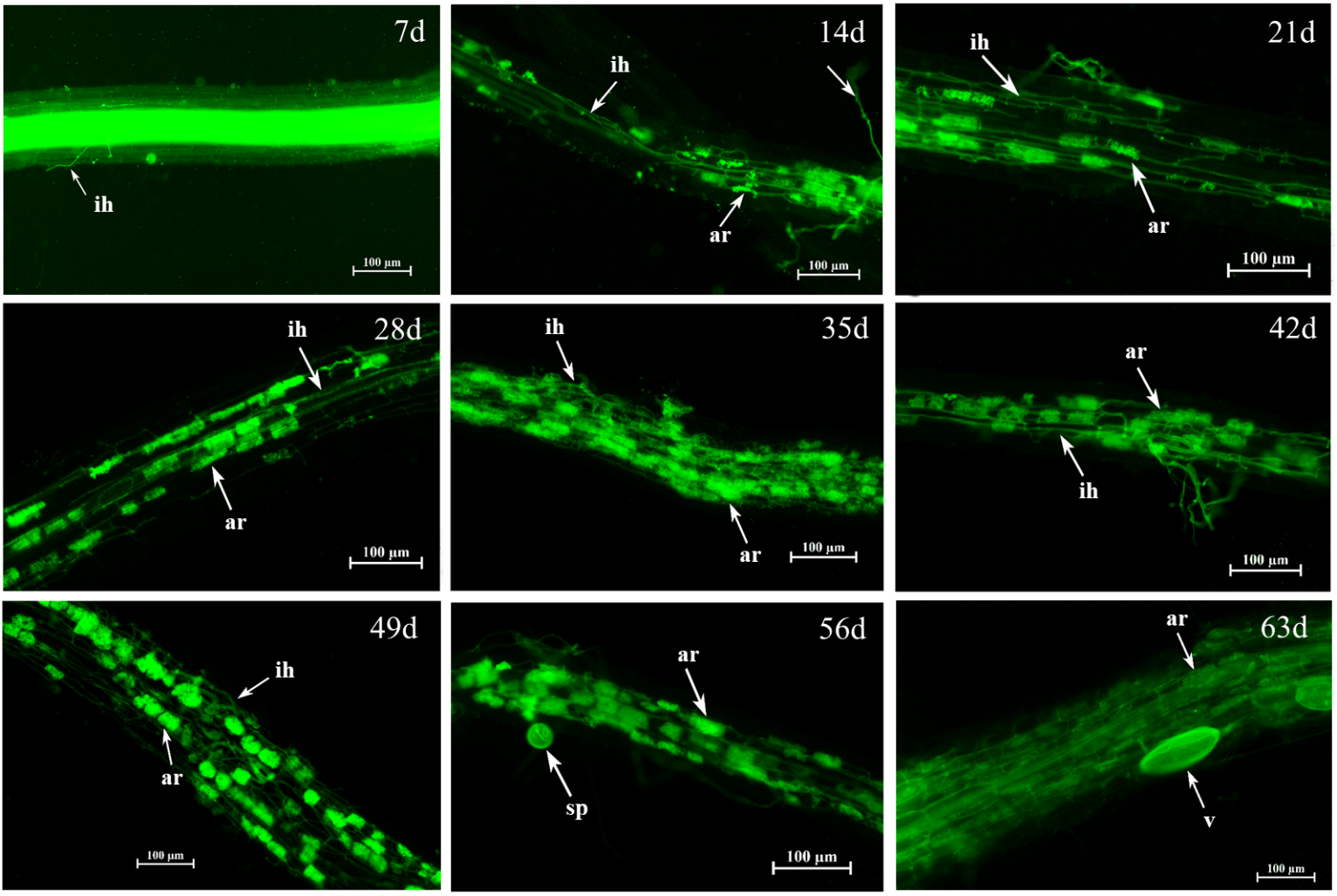


**Fig. S3. The fluorescence images of the morphological structures of *E. grandis* mycorrhizal roots colonized by *R. irregularis* during different stages of AM symbiosis.** The letters in the figures represent different structures of *R. irregularis*: ih, intraradical hyphae; ar, arbuscules; v, vesicles; sp, spores. Scale bars, 100 μm.


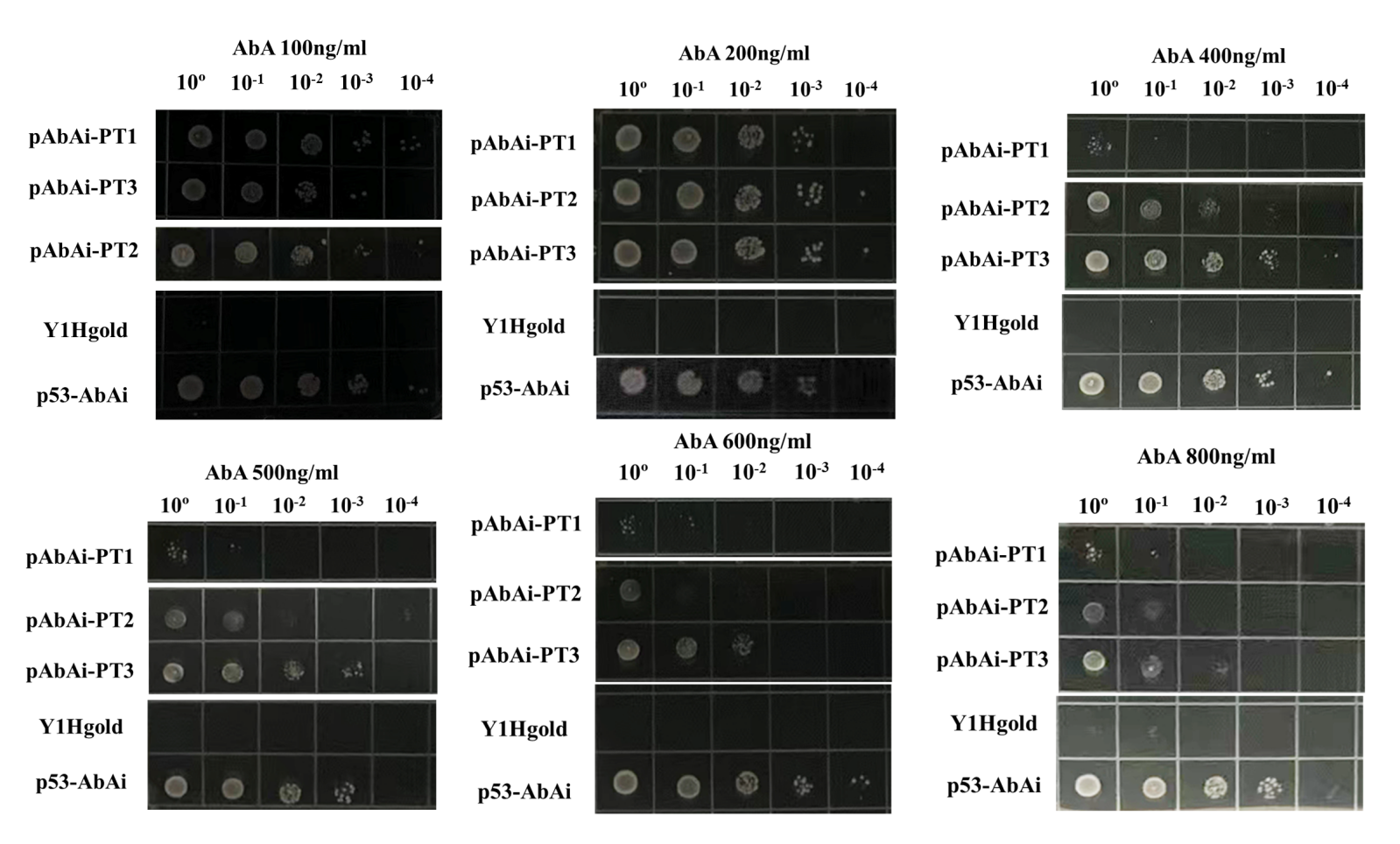


**Fig. S4. Screening of AbA concentrations that pAbAi vectors carrying the P_RiPT1_, P_RiPT2_, P_RiPT3_ promoters could be inhibited on YNB/-Ura solid medium containing 2% glucose for yeast one-hybrid assay.** The p53-AbAi empty vector was transformed into the yeast cells and used as the positive control, while the Y1Hgold empty yeast was used as the negative control.


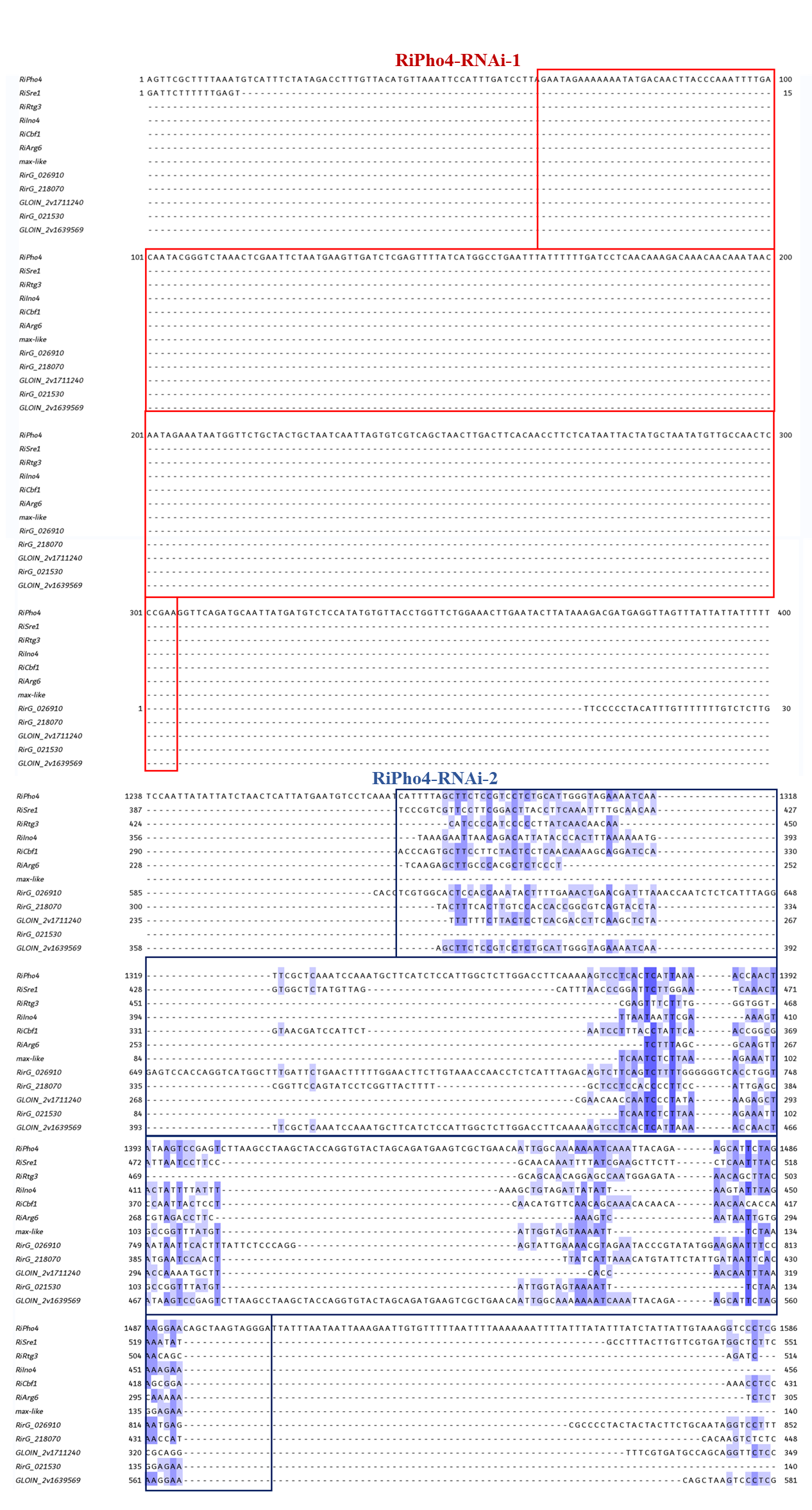


**Fig. S5. Multiple sequence alignments of *RiPho4* and other genes containing bHLH domains in *R. irregularis.*** Silencing region of *RiPho4-RNAi-1* was indicated using a red box, whereas RiPho4-RNAi-2 region was indicated by a blue box. The multiple sequence alignment was performed using Jalview 2.11. The accession numbers were listed in Table S2.

**Table S1. Effect of different phosphate concentrations on the ratio (%) of ACP, ALP and SDH activities and colonization rates of *R. irregularis***

| AMF's colonization | Phosphorus concentrations | ALP activity/colonization （%） | ACP activity/colonization（%） | SDH activity/colonization（%） |
| --- | --- | --- | --- | --- |
| Ratio of active hyphae frequency and colonization frequency (F%) | 30 µM | 98.48±6.62a | 96.13±14.07a | 89.45±13.12a |
|  | 300 µM | 52.25±10.54b | 63.91±10.89bc | 70.03±12.45b |
|  | 1000 µM | 59.89±16.01b | 56.89±13.95b | 77.20±14.80b |
| Ratio of active hyphae intensity and colonization mycorrhizal intensity (M%) | 30 µM | 31.86±15.63c | 67.03±26.73bc | 54.82±24.52bd |
|  | 300 µM | 12.48±5.11d | 29.52±24.76de | 39.14±11.53cd |
|  | 1000 µM | 23.33±13.85d | 27.57±10.80de | 67.01±17.52bd |
| Ratio of active arbuscule abundance and colonization arbuscule abundance (A%) | 30 µM | 19.16±13.61d | 43.43±24.0e | 33.39±15.63c |
|  | 300 µM | 4.85±2.32e | 13.59±5.42d | 30.69±1.41c |
|  | 1000 µM | 9.84±4.39de | 12.67±8.37d | 35.20±10.25c |

Notes: The data shown in the table represent the ratios between enzyme activities and total mycorrhizal colonization determined by enzyme and WGA488 staining, respectively. F%, the colonization frequency; M%, the percentage of mycorrhizal intensity; A%, the percentage of arbuscule abundance. Different letters indicated significant differences between treatments, based on Duncan’s new multi-range test at the alpha=0.05.

**Table S2. The accession numbers of fungal proteins used in this study.**

| Protein names | Accession Numbers | Species |
| --- | --- | --- |
| Rhizophagus-irregularis-RiPho4 | XP_025175129.1 | Rhizophagus irregularis DAOM197198 |
| Lachancea-mirantina-LmPho4 | SCU81032.1 | *Lachancea mirantina* |
| Kazachstania-saulgeensis-KsPho4 | SMN19964.1 | *Kazachstania saulgeensis* |
| Candida-boidinii-CbPho4 | OUM53287.1 | *Candida boidinii* |
| Rhizophagus-diaphanus-RdPho4 | RGB28534.1 | *Rhizophagus diaphanus* |
| Rhizophagus-clarus-RcPho4 | GBB98521.1 | *Rhizophagus clarus* |
| Glomus-cerebriforme-GcPho4 | RIA86088.1 | *Glomus cerebriforme* |
| Gigaspora-margarita-GmPho4 | KAF0357978.1 | *Gigaspora margarita* |
| Gigaspora-rosea-GrPho4 | RIB17793.1 | *Gigaspora rosea* |
| Diversispora-epigaea-DePho4 | RHZ83467.1 | *Diversispora epigaea* |
| Jimgerdemannia-flammicorona-JfPho4 | RUS29008.1 | *Jimgerdemannia flammicorona* |
| Bifiguratus-adelaidae-BaPho4 | OZJ06818.1 | *Bifiguratus adelaidae* |
| Basidiobolus-meristosporus-BmPho4 | ORX93605.1 | *Basidiobolus meristosporus* |
| Candida-glabrata-CgPho4 | XP_445634.1 | *Candida glabrata* |
| Cyberlindnera-jadinii-CjPho4 | CEP25199.1 | *Cyberlindnera jadinii* |
| Saccharomyces-cerevisiae-ScPho4 | NP_116692.1 | Saccharomyces cerevisiae *S288C* |
| Candida-albicans-CaPho4 | AFA36294.1 | *Candida albicans* |
| Aspergillus-thermomutatus-AtPho4 | XP_026611483.1 | *Aspergillus thermomutatus* |
| Funneliformis-mosseae-FmPho4 | CAG8504044.1 | *Funneliformis mosseae* |
| Cetraspora-pellucida-CpPho4 | CAG8510544.1 | *Cetraspora pellucida* |
| Acaulospora-morrowiae-AmPho4 | CAG8545981.1 | *Acaulospora morrowiae* |
| Dentiscutata-heterogama-DhPho4 | CAG8519939.1 | *Dentiscutata heterogama* |
| Claroideoglomus-candidum-CcPho4 | CAG8443911.1 | *Claroideoglomus candidum* |
| Geosiphon-pyriformis-GpPho4 | KAG9295009.1 | *Geosiphon pyriformis* |
| Thamnidium-elegans-TePho4 | KAG2230339.1 | *Thamnidium elegans* |
| Verticillium-dahliae-VdPho4 | AAX73405.1 | *Verticillium dahliae* |
| Histoplasma-capsulatum-HcPho4 | KAG5303002.1 | *Histoplasma capsulatum* |
| Cadophora-malorum-CmPho4 | KAG4418016.1 | *Cadophora malorum* |
| Pyricularia-oryzae-PoPho4 | KAI6629725.1 | *Pyricularia oryzae* |
| Racocetra-persica-RpPho4 | CAG8518643.1 | *Racocetra persica* |
| Acaulospora-colombiana-AcPho4 | CAG8560695.1 | *Acaulospora colombiana* |
| Scutellospora-calospora-SccPho4 | CAG8439467.1 | *Scutellospora calospora* |
| Funneliformis-geosporum-FgPho4 | CAI2170536.1 | *Funneliformis geosporum* |
| Racocetra-fulgida-RfPho4 | CAG8591690.1 | *Racocetra fulgida* |
| Paraglomus-brasilianum-PbPho4 | CAG8490947.1 | *Paraglomus brasilianum* |
| Ambispora-leptoticha-AlPho4 | CAG8509500.1 | *Ambispora leptoticha* |
| Lichtheimia-ramosa-LrPho4 | CDS07329.1 | *Lichtheimia ramosa* |
| Umbelopsis-vinacea-UvPho4 | KAG2181474.1 | *Umbelopsis vinacea* |
| Rhizopus-microsporus-RmPho4 | CEG81708.1 | *Rhizopus microsporus* |
| Radiomyces-spectabilis-RsPho4 | KAI8372717.1 | *Radiomyces spectabilis* |
| Rhizopus-azygosporus-RaPho4 | RCH98832.1 | *Rhizopus azygosporus* |
| Mucor-plumbeus-MplPho4 | KAG2215320.1 | *Mucor plumbeus* |
| Mucor-mucedo-MmPho4 | KAI7873143.1 | *Mucor mucedo* |
| Mucor-saturninus-MsPho4 | KAG2199813.1 | *Mucor saturninus* |
| Rhizopus-arrhizus-RarPho4 | KAG1628845.1 | *Rhizopus arrhizus* |
| Mortierella-polycephala-MpPho4 | KAG0253756.1 | *Mortierella polycephala* |
| Linnemannia-elongata-LePho4 | KAF9305534.1 | *Linnemannia elongata* |
| Podila-humilis-PhPho4 | KAG0333706.1 | *Podila humilis* |
| Podila-epigama-PePho4 | KAF9414635.1 | *Podila epigama* |
| Dissophora-ornata-DoPho4 | KAI8600914.1 | *Dissophora ornata* |
| Lobosporangium-transversale-LtPho4 | XP_021886305.1 | *Lobosporangium transversale* |
| Podila-horticola-PhoPho4 | KAF9317883.1 | *Podila horticola* |
| Mortierella-alpina-MaPho4 | KAF9566829.1 | *Mortierella alpina* |
| Syncephalastrum-racemosum-SrPho4 | ORY94086.1 | *Syncephalastrum racemosum* |
| Podila-clonocystis-PcPho4 | KAG0018257.1 | *Podila clonocystis* |
| Entomortierella-chlamydospora-EcPho4 | KAG0008979.1 | *Entomortierella chlamydospora* |
| RiSre1 | XM_025316076.1 | Rhizophagus irregularis DAOM197198 |
| RiRtg3 | XM_025320094.1 | Rhizophagus irregularis DAOM197198 |
| RiIno4 | XM_025320010.1 | Rhizophagus irregularis DAOM197198 |
| RiCbf1 | XM_025315220.1 | Rhizophagus irregularis DAOM197198 |
| RiArg3 | XM_025310948.1 | Rhizophagus irregularis DAOM197198 |
| RirG_026910 | XM_025320816.1 | Rhizophagus irregularis DAOM197198 |
| RirG_218070 | XM_025314523.1 | Rhizophagus irregularis DAOM197198 |
| GLOIN_2v1711240 | XM_025321956.1 | Rhizophagus irregularis DAOM197198 |
| max-like | XM_025310421.1 | Rhizophagus irregularis DAOM197198 |
| GLOIN_2v1639569 | XM_025318894.1 | Rhizophagus irregularis DAOM197198 |

**Table S3. A list of the primers for qRT-PCR used in this study.**

| GenBank | Genes | Forward primers | Reverse primers |
| --- | --- | --- | --- |
| XM_025318894.1 | *RiPho4* | CTCCACTTTCATTGCACCAAC | TGAAGCATTTGGATTTGAGCG |
| XM_025315184.1 | *RiPT1* | GCACTTATCGTTTTGGCAGC | TCTGGAATTGTGAGACGGAAG |
| XM_025313512.1 | *RiPT2* | GGCGTTTGATACTTGGTTTGG | TGTATATCGTGGCGTTTCTGG |
| XM_025309069.1 | *RiPT3* | GGCGGTGATTATCCTCTATCC | ACAAGAGATACAACTCCAGCG |
| XM_025323230.1 | *RiPT4* | CGCTGGAGTTGTATCTTTTGTTAC | CGAAAATAAAGTGCGATTGTAGGG |
| XM_025317277.1 | *RiPT6* | TGTTGGGTATGGTCTGTGC | CCAATCAACAGCTTTCATGCC |
| XM_025314466.1 | *RiVTC1* | GGAGAGCGACCAAGATAAGAAAG | AATTAGTTACCACGGCACCC |
| XM_025316884.1 | *RiVTC2* | GATGAAGGAGAACAGGAGC | ATGGAAGAGAAGGTGCATGG |
| XM_025316206.1 | *RiVTC4* | TCCAACTACACCAGCCAATG | TGCATCTTCCTCATCTTCGTC |
| XM_025318894.1 | *RiPho4* | CTCCACTTTCATTGCACCAAC | TGAAGCATTTGGATTTGAGCG |
| XM_025320482.1 | *RiPho81* | AAAGCTGTCACAATAGGTACTCC | GATATCATGGTCCTCTTCGCC |
| XM_025321006.1 | *RiPho85* | CCAAGCGACCAATTTCAACC | GTACAGAATCACAAGAGCCCG |
| XM_025309562.1 | *RiPho80* | AGTTACAGCCTCTTCCAAAGC | TTTCCCCAGAGCAGATCAAC |
| XM_025315882.1 | *RiPho2* | TCGACAATATGCTCCGTGAAG | GGGAATAAATTGTGTTGGCGAG |
| XM_025312447.1 | *RiACP1* | GATGGAGACCATGAAGGAGTTAATA | CAGCTACGGAGTACCAAACAA |
| XM_025314866.1 | *RiALP1* | ATAGCCTTGTCACTGATTCCG | GCCAGTCGCTAAACCTAAGTG |
| HM143864 | *RiMSt2* | GGCAGGATATTTGTCTGATAG | GCAATAACTCTTCCCGTATAC |
| XM_025321412.1 | *RiEF1α* | TGTTGCTTTCGTCCCAATATC | GGTTTATCGGTAGGTCGAG |
| XM_025316906.1 | *RiPPN1* | TGCACAACATATCCCACCTT | TCCAGATTTGTGTGACCAGTAA |
| XM_025331091.1 | *RiPPX1* | AGACGAAAGATTATATCTCAACATCAAG | TGGCTAATCTAAAATCATCTCGGG |
| CBMM010022766.1 | *NbPT4* | CTCCCCATTTTATCTCCGAGC | TGTAAATGGAGTCGCCTTCTG |
| ON012824 | *EgPT4* | TGATCCTTTCCGCACTGTTC | ATCCGCTTCTGGTTGTGTAG |
| ON012824 | *EgUBI3* | TCACCTACGTCTACCAGAAGG | TCCTCGAAAGCTGTAAACATGG |

**Table S4. A list of the primers for functional analysis of *RiPho4***

| Primer names | Primer sequences (from 5’ to 3’) | Use for |
| --- | --- | --- |
| *pUG36-RiPho4-F* | AGAACTAGTGGATCCATGACAACTTACCCAAATTTTG | Subcellular localization in yeast |
| *pUG36-RiPho4-R* | CAGCCCGGGGGATCCTTAACATTCAGTGGGAGAAGTTG | Subcellular localization |
| *ADRiPho4-F* | GGAGGCCAGTGAATTCATGACAACTTACCCAAATTTTG | Yeast one-hybrid |
| *ADRiPho4-R* | CGAGCTCGATGGATCCTTAACATTCAGTGGGAGAAGTTG | Yeast one-hybrid |
| *VigsPho4-1F* | GGGGCTCTAGAAGGCCTCCATGGGGAAAAAAATATGACAACTTACCC | Virus-induced gene silencing |
| *VigsPho4-1R* | GGGGCGCGTGAGCTCGGTACCGGATCCGGGAGTTGGCAACATATTAGC | Virus-induced gene silencing |
| *VigsPho4-2F* | GGGGCTCTAGAAGGCCTCCATGGGCATTTTAGCTTCTCCGTCCTC | Virus-induced gene silencing |
| *VigsPho4-2R* | GGGGCGTGAGCTCGGTACCGGATCCTCCCTACTTAGCTGTTCCTTCTAG | Virus-induced gene silencing |
| *RiPT1-F* | AGCACATGCCTCGAGGTCGACGATGTATCTGATAGTG | Yeast one-hybrid |
| *RiPT1-R* | GGTACCCGGGGATCTGTCGACTCTACCAACCAAGGC | Yeast one-hybrid |
| *RiPT2-F* | AGCACATGCCTCGAGGTCGACCCATTTAATTTATGATTAG | Yeast one-hybrid |
| *RiPT2-R* | GGTACCCGGGGATCTGTCGACGATACCAATGATGGCAAG | Yeast one-hybrid |
| *RiPT3-F* | AGCACATGCCTCGAGGTCGACGGCTACTTTGTGTCTTCATCAG | Yeast one-hybrid |
| *RiPT3-R* | GGTACCCGGGGATCTGTCGACGGGTGATCACTTAGTACAACAGGGG | Yeast one-hybrid |
| *RiPT1-dF1* | AAATAAAGGACGTTTGTTACC | Site-specific mutagenesis |
| *RiPT1-dR2* | GGCCGGAAAAAATATTTTATC | Site-specific mutagenesis |
| *RiPT1-dF2* | GATAAAATATTTTTTCCGGCC | Site-specific mutagenesis |
| *RiPT2-dR1* | CGCATATATATTATAAATTTG | Site-specific mutagenesis |
| *RiPT2-dF1* | CAAATTTATAATATATATGCG | Site-specific mutagenesis |
| *RiPT2-dR2* | GTCATTTAGTTCATTGG | Site-specific mutagenesis |
| *RiPT2-dF2* | CCAATGAACTAAATGAC | Site-specific mutagenesis |
| *RiPT3-dF* | AGCACATGCCTCGAGGTCGACCATCTCGTTGATTTAGAAAGTTTCGGG | Site-specific mutagenesis |
| *RiPT3-dR* | GGTACCCGGGGATCTGTCGACCTACAATCATCATTATTCTGTTAAGGG | Site-specific mutagenesis |

**Table S5. RiPho4-binding sites of the PHO pathway downstream gene promoters**

| Genes | Motif | Sites | | | |
| --- | --- | --- | --- | --- | --- |
| *RiPT1* | CACGTG/T | -2563F | -2722F |  |  |
| *RiPT2* | CACGTG | -2537F | -2563F |  |  |
| *RiPT3* | CACGTG | -1188F | -976F |  |  |
| *RiPT4* | CACGTT | -708F |  |  |  |
| *RiPT6* | CACGTG/T | -655F | -2134F | -1739F |  |
| *RiALP1* | CACGTG/T | -78F | -302F | -1035F | -2741F |
| *RiVTC1* | CACGTT | -83F |  |  |  |
| *RiVTC2* | CACGTG | -599F |  |  |  |

Notes; ‘F’ refers to the translation start codon ATG of the DNA fragment, ‘-’ represents the upstream of the ATG, and the numbers before letters represent the number of bases from the position of Pho4-binding site of promoters to the initiation codon.
